# Efficient tribological properties of azomethine-functionalized chitosan as a bio-lubricant additive in paraffin oil: experimental and theoretical analysis†

**DOI:** 10.1039/d0ra07011d

**Published:** 2020-09-10

**Authors:** Manilal Murmu, Sirsendu Sengupta, Ritam Pal, Sukdeb Mandal, Naresh Chandra Murmu, Priyabrata Banerjee

**Affiliations:** Surface Engineering and Tribology Division, Central Mechanical Engineering Research Institute M.G. Avenue, City Centre Durgapur 713209 West Bengal India pr_banerjee@cmeri.res.in priyabrata_banerjee@yahoo.co.in www.priyabratabanerjee.in https://www.cmeri.res.in; Academy of Scientific and Innovative Research CSIR-Human Resource Development Centre (CSIR-HRDC) Campus, Postal Staff College Area, Sector 19, Kamla Nehru Nagar Ghaziabad 201 002 Uttar Pradesh India; Department of Mechanical Engineering, Jadavpur University Jadavpur Kolkata 700032 West Bengal India

## Abstract

A simple condensation of chitosan (from shrimp shells) and 4-hydroxybenzaldehyde was performed to yield bio-lubricant additive comprised of azomethine functional groups to be used with paraffin lube oil in industries. The synthesized Schiff base derivative of chitosan (SBC) additive was characterized using a CHN analyzer and FT-IR spectroscopy, and the thermal stability was explored using thermogravimetry. The rheological properties of SBC additives in paraffin oil were studied and are discussed herein. The tribological properties of SBC were tested in paraffin as the base oil employing a four-ball tester with different experimental conditions (*viz.* the concentration of the additive, applied load, speed and time duration), following ASTM D4172A standards. The optimum concentration of the additive in the base oil was found to be 150 ppm, exhibiting minimum coefficient of friction, but with higher concentrations of additive in base oils, the coefficient of friction increased. UV-Vis spectroscopy studies were also performed to confirm the formation of SBC and dispersion stability. The determined tribological parameters, such as the coefficient of friction, mean wear scar diameters and mean wear scar volumes, were found to significantly reduce the coefficient of friction of paraffin oil upon the addition of SBC. The state of steel balls upon exposure to various experimental conditions was analyzed and explained based on outcomes from FESEM, EDX, ferrography and AFM spectroscopy. The insights into interactions of the synthesized SBC with the metal surface were explored using *ab initio* density functional theory, Fukui indices, molecular dynamics simulation and radial distribution function.

## Abstract 2

Schiff base derivative of chitosan as biolubricant additive explored in paraffin lube oil.

## Graphical abstract

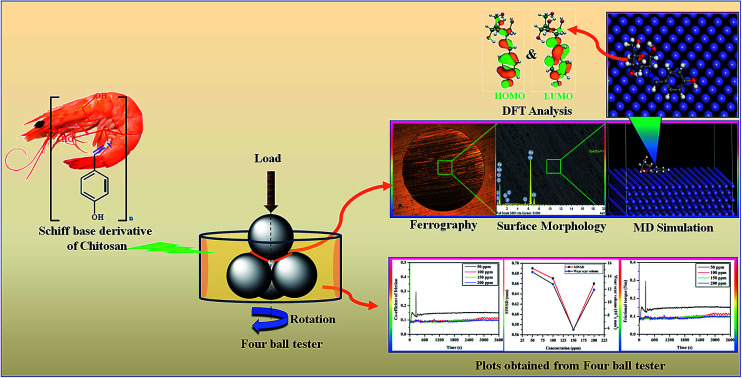

## Introduction

Energy dissipation between moving parts of a machine must be reduced to achieve an appreciable efficiency. Further, the reduction in friction due to application of lubricants lowers the energy dissipation. Low friction and good reliability of a machine ensure economic performance and protection of the environment. Lubricants play a crucial role in reducing the friction between contact surfaces so that the longevity and performance of machinery components are increased as desired. Lubricant additives such as engine oils, transmission oils, hydraulic oils, fishing, and chainsaw oils are used in several industries including automobile, mining, and agriculture. The lubricants must possess characteristic properties such as chemical stability, viscosity, fluidity, solubility, workability in different ranges of temperatures, and flammability.^1^ The liquid lubricants are mainly based on mineral oils, polyol esters, perfluoropolyethers, polyalphaolefins, *etc*.^2^ The demand for lubricants is increasing with the growth of industries but the depletion of crude oil has become an important concern for lubricant manufacturers since major lubricant synthesis depends on crude oils.^3,4^ However, fresh lubricants, as well as used lubricants, may cause considerable damage to the environment. Moreover, water and soil are affected by the disposal or leakage of lubricants, and air is also polluted by volatile lubricants.^1,5^ In general, a lubricant is comprised of base oil and an additive. The essential property of a lubricant is its thin film-forming capability between the contact surfaces so that the wear on surfaces is reduced.^6–9^ The wear-reducing behaviour and its characteristic tribological properties are primarily influenced by the application of thickeners, as well as lubricant additives. The base oil requires improvement by incorporating characteristic corrosion inhibition properties so that it can find applications in several industries.^10^ The additive concentration ranges from 10–20% of the lubricant. Moreover, additives that are present in the lubricant can be toxic to flora and fauna. Zinc dialkyldithiophosphates have been constantly used as additives in lubricants to prevent wear,^11–15^ but as the sulfur and phosphorous contents are high, the additive may be harmful to the environment and hence, their use has to be limited.^16–19^ Several studies have reported that petroleum-based lubricants are harmful to human health. Dermal exposure to petroleum-based lubricants may cause damage to the respiratory system, and can also cause cancer.^1,20–22^ Furthermore, the degradation of petroleum-based lubricants is more harmful as this increases their toxicity; hence, the protection of the environment has become a primary concern in recent times. In this regard, the authorities are still involved in formulating and enacting strict measures to minimize the application of lethal additive ingredients in industrial products in the field of lubricants.^3,23,24^ In this context, it has recently been reported that bio-lubricants are biodegradable, non-toxic and pose little risk to the environment.^1,25,26^ Bio-lubricants also enhance performance, including better lubricity, low volatility and compressibility, high viscosity, shear stability and resistance towards humidity.^1,27–32^ Consequently, bio-lubricants have emerged as an important topic of research in recent times.

Chitosan is obtained by the partial or full deacylation of the most abundant biopolymer, chitin, which is a natural polysaccharide synthesized by a large number of living organisms and it is mostly found in the exoskeletons of crustaceans such as shrimps, crabs and lobsters.^33,34^ Chitosan consists of 2-*N*-acetyl-2-deoxyglucose(*N*-acetylglucosamine) and 2-amino-2-deoxyglucose(glucosamine) units linked with β-1,4-linkages in its skeleton. It is considered as a biocompatible, biodegradable and non-toxic natural polymeric material that also has inherent antioxidant activity. These characteristic properties of chitosan make it suitable for vast applications, including waste treatment, chromatography, cosmetics, textiles, photographic papers, biodegradable films, biomedical devices, drug delivery agents, and in the food industry such as antimicrobial, emulsifying, thickening and stabilizing agents.^2,35–39^

On the other hand, it is known that Schiff bases have better load-carrying capabilities, anti-corrosion and anti-wear properties, which facilitate their application as additives for lubricating greases.^40,41^ Recently, it has been observed that there is scant literature revealing the lubricity properties of Schiff base derivatives in paraffin oil.^42–49^ Motivated by this, the present work has been carried out to explore and explain the anti-wear properties of a bio-benign lubricant additive and investigate the insights into its adsorption mechanism on the metal substrate, as well as to correlate the experimental findings with the theoretical calculations. In this regard, an azomethine-functionalized derivative of chitosan (SBC) has been synthesized using the Schiff base condensation reaction of chitosan and 4-hydroxybenzaldehyde. The novelty of the work is the application of the azomethine-functionalized derivative of chitosan (SBC) as a lubricant additive. It has been revealed that the minimum concentration (herein, 150 ppm) of SBC in paraffin oil exhibits better results. The experimentally obtained results correlate well with *ab initio* density functional theory, Fukui indices and molecular dynamics simulation, along with the analysis of the radial distribution function. The theoretical studies provided insightful explanations for the adsorption of the SBC on metal surfaces leading to the tribo-film formation and the consequent reduction in the coefficient of friction.

## Experimental details

### Materials

Chitosan (from shrimp shells) and 4-hydroxybenzaldehyde used in this work were purchased from Sigma Aldrich, whereas, methanol, glacial acetic acid, acetone and paraffin liquid light oil were purchased from Merck India. The chitosan is >75% deacetylated. The paraffin liquid light was the lubricating base oil with a specific gravity of 0.830 at 20 °C and kinematic viscosity of 30 cSt at 37.8 °C. The approximate elemental composition of chrome steel balls used in this experiment was 1.30–1.60% Cr; 0.95–1.10% C; 0.25% Mn; 0.15–0.35% Si; 0.03 P%; 0.025% S and the rest is iron.

### Azomethine (Schiff base) functionalization of chitosan

**Scheme 1:**
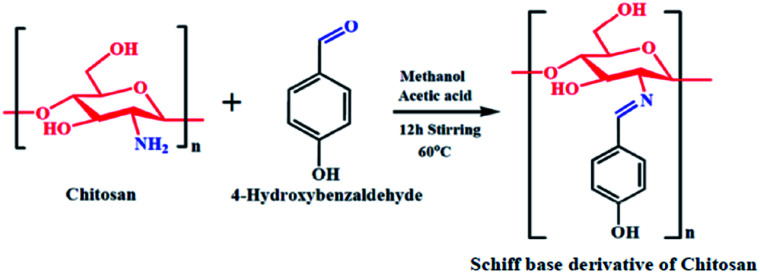
Schematic representation of the synthesis of the Schiff base derivative of chitosan (SBC).

Chitosan (3.5 mmol) was added to methanol (30 ml) in a dry round bottom flask. The mixture was allowed to stir for 30 minutes to obtain a homogeneous emulsion. Next, 4-hydroxybenzaldehyde (10.5 mmol) was taken in a beaker and 5 ml of methanol was added, followed by 1 ml of glacial acetic acid dropwise and the mixture was stirred for 12 hours at 60 °C; an orangish-yellow precipitate was obtained. At the end of stirring, the orangish-yellow precipitate was filtered and dried in a desiccator at room temperature. The obtained precipitate was the azomethine functionalized chitosan, *i.e.*, the Schiff base derivative of chitosan (SBC). A schematic diagram representing the synthesis of SBC has been given in Scheme 1.

### Characterization of the Schiff base derivative of chitosan

The synthesized compounds were characterized using a CHN analyzer (Perkin Elmer 2400C elemental analyzer). FT-IR spectroscopy was conducted using an FT-IR Spectrum 100 spectrometer (Perkin Elmer), and the ^1^H-NMR characterizations of chitosan and Schiff base derivative of chitosan (SBC) are described in Fig. S1 in the ESI.† The FT-IR spectra were recorded using KBr pellets. The thermal stability was explored using thermogravimetry (NETZSCH TG 209F3). Thermogravimetric analysis was performed using an Al_2_O_3_ crucible with a temperature ramp of 10 K min^−1^ in an inert nitrogen atmosphere for a temperature range of 30 °C to 900 °C.

### Tribology test

Before performing the tribological tests, a homogeneous solution of SBC in paraffin oil was prepared by ultrasonication for 1 h. The images of the homogeneous dispersion of SBC in paraffin oil are shown in Fig. S2 in the ESI.† The dispersion stabilities of the different concentrations of SBC in paraffin oil were analyzed using an Agilent UV-Vis spectrometer (Cary 60). The coefficient of friction was measured using a four-ball tester (Ducom, India) as per the ASTM D4172A standard. Subsequently, ferrography was used to evaluate the other tribological properties such as wear scar diameter (WSD), mean wear scar diameter (MWSD) and wear scar volume. Different sets of four 12.7 mm diameter steel balls were used to execute these experiments. Among the four balls, one ball was placed at top of the horizontally placed three immobile balls held tightly in a sample holder, so that it could be subjected to rotation against the fixed balls under the applied load. Different concentrations of lubricant formulations were prepared by adding 50 ppm, 100 ppm, 150 ppm and 200 ppm of the synthesized SBC additive in the lubricating base oil (herein liquid paraffin light oil). The admixtures were thoroughly ultrasonicated to obtain the homogeneous formulation. The as-prepared lubricant formulations were applied to the clamped sample holder and the four steel balls were kept immersed during the experiment. The experiments were carried out using a 392 N load with 1200 rpm rotating speed for 3600 seconds at 75 °C as per the ASTM D4172A standard. The optimum SBC additive concentration in the base lube oil yielding the desired lubricity was identified (herein 150 ppm SBC in the base lube oil). Thereafter, with this optimum concentration of SBC additive in paraffin oil, all the tests were carried out by varying the load, speed and time, according to the ASTM standard. The load was varied as follows: 196 N, 294 N, 392 N and 490 N at a constant speed of 1200 rpm at 75 °C for 3600 seconds. Again, the speed was varied from 800 rpm, 1000 rpm, 1200 rpm and 1400 rpm at a constant load of 392 N at 75 °C for 3600 seconds. Furthermore, the experiment run time was varied as follows: 900 seconds, 1800 seconds, 2700 seconds and 3600 seconds at a constant load of 392 N at the speed of 1200 rpm at 75 °C. With all these test samples, the tribological properties such as coefficient of friction, mean wear scar diameters and wear scar volumes were determined.

### Mean wear scar diameter

The diameters of the worn-out steel balls were ascertained by carefully observing them under a microscope equipped with two screw gauges at right angles to each other. These screw gauges were used to measure two diameters, *viz.* the sliding diameter (*d*_s_) and perpendicular diameter (*d*_p_) on the same ball. The geometric mean of the two diameters was considered to calculate the mean of the wear scar diameter for each of the three balls as follows:1
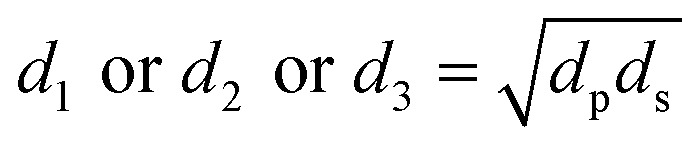


For each run, the mean wear scar diameter (MWSD) was represented as *d*, the arithmetic mean of the diameters of the three balls (*d*_1_, *d*_2_ and *d*_3_) as follows:2
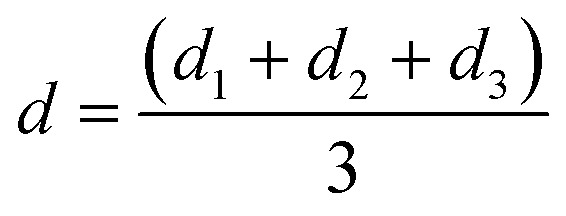
where, *d*_1_ or *d*_2_ or *d*_3_ are the wear scar diameters of each worn-out ball, while, *d* refers to the mean wear scar diameter for that particular set of experiments.

**Scheme 2:**
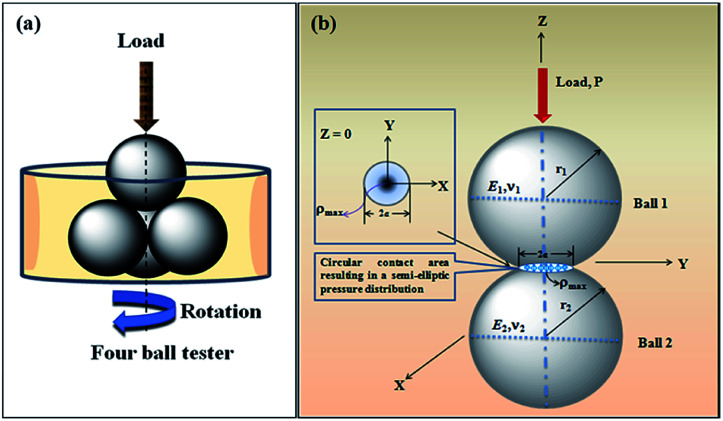
(a) Representational image showing four balls placed in a sample holder. (b) Contact between two interacting balls in a four-ball tester.

The contact between two spherical balls is diagrammatically shown in Scheme 2. Ball 1 is the upper ball and Ball 2 represents the three horizontally placed balls. The load is applied through the upper ball towards the lower balls. The rotating upper ball under pressure generates scars on the surfaces of the horizontally placed balls that are in contact with it.

### Mean wear volume

The mean wear volume (*V*) is another important tribological parameter to be considered. The mean wear volumes for the worn-out steel balls were measured using the mean wear scar diameters as follows:^47,48^3
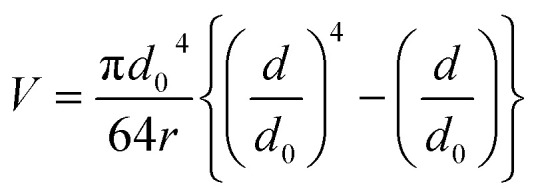
where *d* is the MWSD, the effective radius of the two contacting balls is *r*, and *d*_0_ represents the contact diameter between two steel balls, *i.e.*, the Hertzian diameter as shown in Scheme 2, which is determined by using the following equation:^50,51^4
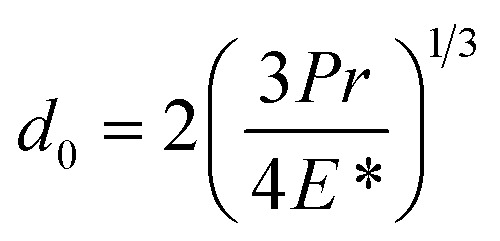
*P* is the actual load measured in N on each of the three horizontal balls, which is 0.408 times the applied load; *r* refers to the effective radius of two steel balls (Ball 1 and Ball 2) of radius *r*_1_ and *r*_2_, respectively; E* is the resultant modulus of elasticity calculated from eqn (5) and (6), respectively.5
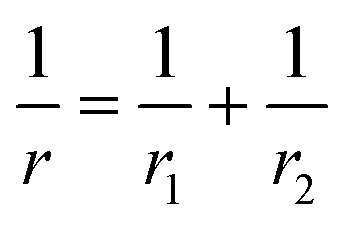
*r*_1_ and *r*_2_ are the radii of the upper and the lower steel balls, respectively.6
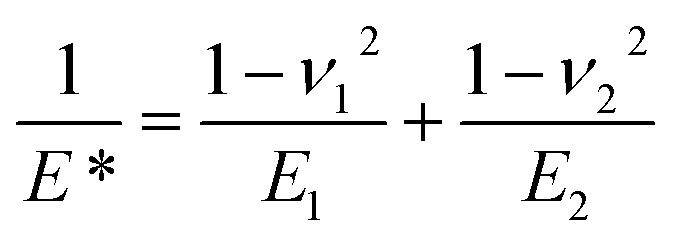
*ν* is the Poisson ratio, in this case *ν*_1_ = *ν*_2_ = 0.3; *E*_1_ = *E*_2_ = 206 GPa, *ν*_1_ and *ν*_2_ refer to the Poisson ratio of the upper (Ball 1) and lower steel ball (Ball 2), while, *E*_1_ and *E*_2_ refer to the modulus of elasticity of the upper (Ball 1) and lower steel balls (Ball 2), respectively.

Furthermore, the contact diameter (*d*_0_) is twice the radius (*a*) of the circular contact area and it was determined as follows:^50,51^7
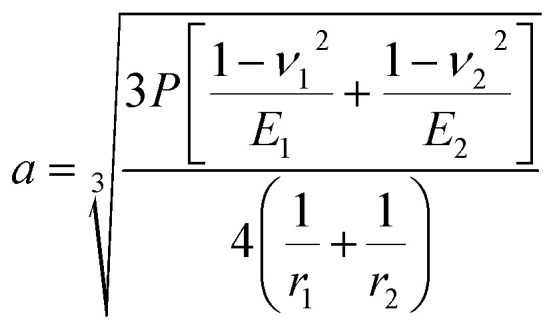


The maximum contact pressure (*ρ*_max_) at the centre of the circular contact area is given as follows:^49^8
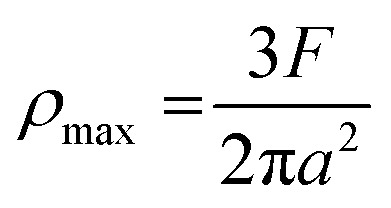


The depth of indentation (*d*_i_) is related to the maximum contact pressure as follows:^52^9
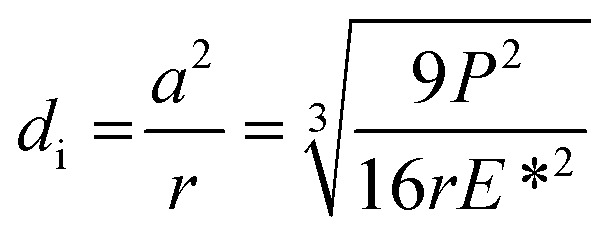
where *P* represents the actual load on each of the horizontal stationary balls.

The maximum shear stress (*τ*_max_) occurring in the interior of the balls was ∼0.49*a* for *ν* = 0.3.

### Rheological study

The rheology study of a homogeneous solution of varying concentrations of SBC additive in paraffin oil was investigated using an Anton Paar Physica MCR-501 rheometer. A parallel plate geometry with a fixed lower plate of 40 mm diameter. The rotating upper plate was placed at a gap of 1 mm and 5 mm for the sample temperatures set at 25 °C and 75 °C, respectively, used under controlled shear stress conditions with an accuracy of ±0.01 °C. The dynamic viscosity was measured at a shear rate of 100 s^−1^.

### Surface morphologies

The surfaces of the worn-out steel balls retrieved after the experiment in the lubricant solution with different concentrations of SBC additive in paraffin oil were analyzed using Field Emission Scanning Electron Microscopy (FESEM) of Sigma HD Zeiss, Germany and Ferrogram maker and wear particle Analyser (Analytical Ferrograph, model-T2FM, Spectro Inc. Industrial Tribology Systems, Massachusetts, US). The tribo-film composition on the worn-out steel balls was analyzed using an energy-dispersive X-ray spectrometer (EDX) equipped with FESEM. The surface topography of the worn-out surface of the steel balls was analyzed using an atomic force microscope (AFM) of Nanosurf C3000, Switzerland.

### Theoretical calculations

The theoretical analysis of the molecular or electronic properties and the insightful exploration revealing the interaction capability of organic molecules with metal surface atoms are very necessary to correlate the obtained experimental results.^53–55^ As such, *ab initio* quantum chemical calculations through density functional theory, local reactivity sites recognition through Fukui indices analysis, simulated interactions of the molecule with the metal surface atoms using molecular dynamics simulation, and radial distribution function analysis have been carried out in the present work.

### Quantum chemical calculations

The density functional theory (DFT) calculations based on quantum chemical calculations were performed using a highly efficient ORCA programme package, version 2.7.0, developed by Prof. Frank Neese.^56^ The optimizations of the geometry and exchange–correlation calculations were carried out by using the hybrid B3LYP functional, which considers Becke's three-parameter exchange functional with the Lee–Yang–Parr nonlocal correlation functional.^57^ Triple zeta quality TZV (P) basis set for N and O atoms and the polarized split valence SV (P) basis set for C and H atoms of all-electron Gaussian basis sets were used.^58,59^ The self-consistent fields (SCF) were converged with 10^−7^ Eh density change, 10^−8^ Eh energy and the maximum element of the Direct Inversion of/in the Iterative Subspace (DIIS) error vector of 10^−7^.

### Fukui indices analysis

Generally, the organic molecules interact with metal surface atoms through donor–acceptor (D–A)-type interaction. Herein, the most reactive atoms of the molecule participate in the D–A-type interaction. The Fukui indices (FIs) were also analyzed to identify the presence of locally reactive atoms in the skeleton of the organic molecule. This was achieved by employing the DMol^3^ Module available within the Material Studio™ version 6.1,^60^ utilizing the double numerical polarization (DNP) basis set with the aim to include both p and d orbital polarization functionals. The DNP basis set is associated with the generalized gradient approximation (GGA) and Becke–Lee–Yang–Parr (BLYP) exchange–correlation functional.^61^

### Molecular dynamics simulation

The interactions of organic molecules with metal surface atoms can be easily explored by employing molecular dynamics (MD) simulation. The adsorption propensity and actual mode of adsorption of the targeted molecule can be effectively obtained using MD simulation. In this experiment, Material Studio™ version 6.1 was employed for modelling a three-dimensional simulation box with dimensions of 37.90 Å × 37.90 Å × 69.72 Å and executing the simulation. Herein, the required simulation box was modelled using 10 layers of iron atoms with 110 planes as the base slab so that the surface depth and the corresponding cut off for radius is maintained. The iron 110 plane, *i.e.*, Fe (110) was chosen as it is comprised of a highly packed structure as well as it is energetically highly stable;^62,63^ thereafter, the targeted organic molecule was placed above it. It is noteworthy that the topmost slab was kept empty or considered as a vacuum slab. The coordinates of the base, *i.e.* the entire iron layer, were frozen for case one, and in another case, the bottom eight layers were frozen and considered as the bulk layer keeping the upper two layers relaxed and allowing them to freely interact with the organic molecule during dynamics simulation.^64,65^ The constructed simulation box was then subjected to optimization using the condensed phase optimized molecular potential for atomistic simulation (COMPASS) force field. The COMPASS force field is highly efficient in predicting the chemical properties of various molecules. MD simulation results were derived by employing fine quality dynamics with the NVT canonical ensemble in a Brendsen thermostat with a time step of 1.0 fs for the simulation time of 200 ps at 298 K. After completion of the MD simulation, complete insight into how the molecule interacts with the iron atoms was gained. The interaction energies for the entire simulation box, the energy of the interacting surface, as well as the individual energies of the studied molecules were determined.

The energy of interaction (*E*_interaction_) was determined using the following equation:

10 *E*_interaction_ = *E*_total_ − (*E*_surface_ + *E*_molecule_)

where, *E*_total_ denotes the total energy for the entire system, *E*_surface_ represents the energy of the surface and *E*_molecule_ refers to the energy of the studied organic molecule. The obtained interaction energy was used to determine the binding energy as follows:

11 *E*_binding_ = −*E*_interaction_

## Results and discussion

### Characterization of the Schiff base derivative of chitosan

**Fig. 1:**
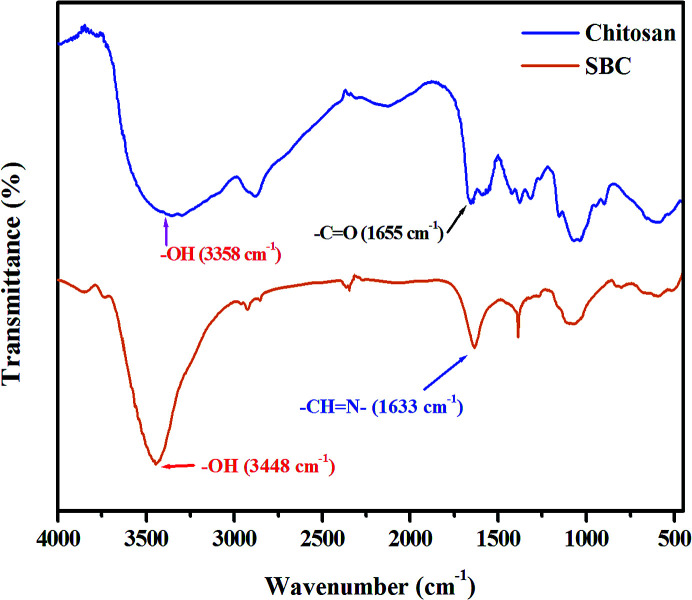
FT-IR peak of chitosan and the Schiff base derivative of chitosan (SBC).

The characterization of the Schiff base derivative of chitosan (SBC) is discussed as follows. FT-IR spectra of SBC were compared with that of chitosan. Zhang *et al.* reported that the –OH stretching band in chitosan is between 3450 cm^−1^ and 3100 cm^−1^. The peak at 1381 cm^−1^ represents the –C–O stretching of the primary alcoholic group. The other major peaks between 1250 cm^−1^ and 1085 cm^−1^ were attributed to the –NH_2_ group.^66^ The FT-IR spectrum of SBC has been shown in Fig. 1, where it can be observed that the peak of the azomethine functional group ( <svg xmlns="http://www.w3.org/2000/svg" version="1.0" width="10.400000pt" height="16.000000pt" viewBox="0 0 10.400000 16.000000" preserveAspectRatio="xMidYMid meet"><metadata> Created by potrace 1.16, written by Peter Selinger 2001-2019 </metadata><g transform="translate(1.000000,15.000000) scale(0.011667,-0.011667)" fill="currentColor" stroke="none"><path d="M80 1160 l0 -40 40 0 40 0 0 -40 0 -40 40 0 40 0 0 -40 0 -40 40 0 40 0 0 -40 0 -40 40 0 40 0 0 -40 0 -40 40 0 40 0 0 -40 0 -40 40 0 40 0 0 -40 0 -40 40 0 40 0 0 80 0 80 -40 0 -40 0 0 40 0 40 -40 0 -40 0 0 40 0 40 -40 0 -40 0 0 40 0 40 -40 0 -40 0 0 40 0 40 -40 0 -40 0 0 40 0 40 -80 0 -80 0 0 -40z M560 520 l0 -40 -40 0 -40 0 0 -40 0 -40 -40 0 -40 0 0 -40 0 -40 -40 0 -40 0 0 -40 0 -40 -40 0 -40 0 0 -40 0 -40 -40 0 -40 0 0 -40 0 -40 -40 0 -40 0 0 -40 0 -40 80 0 80 0 0 40 0 40 40 0 40 0 0 40 0 40 40 0 40 0 0 40 0 40 40 0 40 0 0 40 0 40 40 0 40 0 0 40 0 40 40 0 40 0 0 80 0 80 -40 0 -40 0 0 -40z"/></g></svg> CH=N–) of SBC is present at ∼1633 cm^−1^. The peaks of –OH and –C–O–C were also evident at ∼3448 cm^−1^ and ∼1086 cm^−1^, respectively, showing the inherent peaks present in chitosan, but no peak of the –NH_2_ group. This suggests the utilization of the –NH_2_ group during the Schiff base condensation reaction. Thus, the formation of the azomethine linkage between the amine group of the d-glucosamine moiety of chitosan and the aldehyde group of 4-hydroxybenzaldehyde yielding the Schiff base derivative of chitosan (SBC) was confirmed.

### Determination of the degree of azomethinization

The azomethine functionalization of chitosan was performed to obtain the product SBC. Assuming that no loss occurred during the synthesis and workup of getting the SBC product, it was found that 0.1 g of chitosan yielded 0.1191 g of SBC. This suggests that there was a weight gain of ∼19% which is higher than the chitosan monomer unit. The molecular weight of the monomer unit, *i.e.*, (C_6_H_11_O_4_N)_0.75_–(C_8_H_13_O_5_N)_0.25_, is 171.50 g mol^−1^. As reported by Singh *et al.*,^67^ the molecular weight of the SBC monomer unit having the empirical formula (C_6_H_11_O_4_N)_0.75−*x*_–(C_8_H_13_O_5_N)_0.25_–(C_13_H_15_O_5_N)_*x*_ is expected to be 204.08 g mol^−1^, where *x* is the degree of azomethinization (∼31.33%). The results obtained from the CHN analysis of (C_6_H_11_O_4_N)_0.4367_–(C_8_H_13_O_5_N)_0.25_–(C_13_H_15_O_5_N)_0.3133_ are as follows: (analytically calculated) C: 58.86; H: 5.70, N: 5.28 and O: 30.16. Found: C: 60.89; H: 5.87, N: 5.63; O: 27.61. The CHN analysis of the SBC additive confirmed its formation.

### Thermal stability analysis

**Fig. 2:**
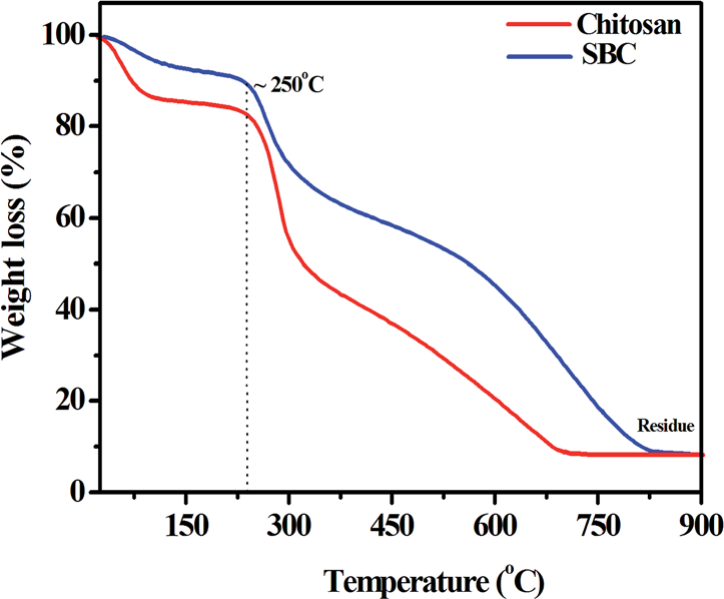
Thermogravimetric curve of the chitosan and Schiff base derivative of chitosan (SBC) in a nitrogen atmosphere with a heating rate of 10 °C min^−1^.

The thermal stabilities of chitosan and SBC have also been studied and are presented in Fig. 2.

An initial weight loss up to 100 °C was observed, which may be attributed to the evaporation of trapped moisture within the chitosan and the SBC compound. It was also noticed that the degradation, *i.e.*, disruption of the structures of both chitosan and SBC was initiated after 250 °C, which may be due to the dehydration of the saccharide ring or the depolymerisation reaction. Fig. 2 also reveals the abrupt degradation of chitosan as compared to that of the modified chitosan (*i.e.*, SBC). This may be attributed to the incorporation of azomethine linkages and the aromatic moiety in chitosan. The degradation temperature of SBC was slightly less than that of chitosan, which may be attributed to the loosening of the packing structure by the introduction of functional groups as reported by Singh *et al.*^67^ Finally, the degradation of chitosan and SBC was complete at ∼690 °C and ∼820 °C, respectively, with a residual mass of approximately 8%. Since the compound has significant thermal stability, it proves to be a good choice for the lubricant additive.

### Dispersion stability analysis

**Fig. 3:**
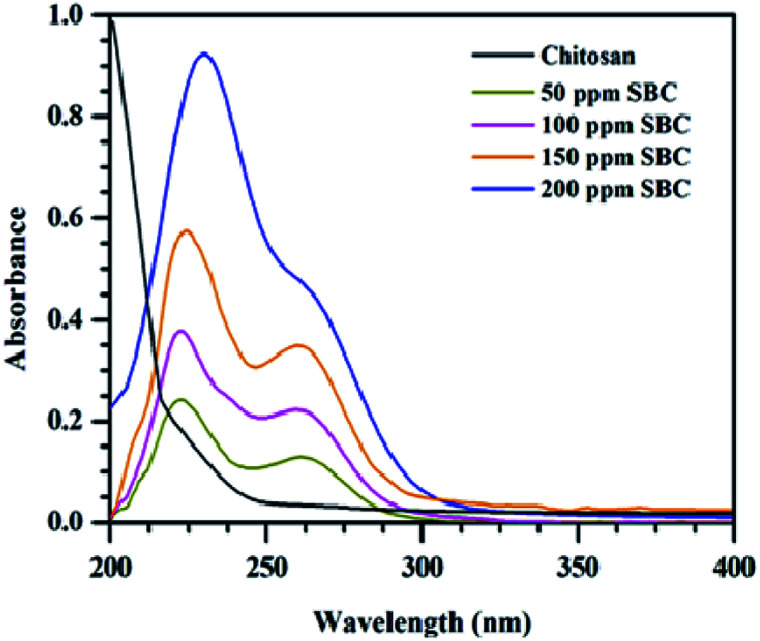
UV-Vis spectra of chitosan and the Schiff base derivative of chitosan (SBC).

The dispersion stability of different concentrations of SBC in paraffin oil was analyzed using UV-Vis spectrometry techniques. The UV-Vis spectra of different concentrations of SBC additives added in paraffin oil are shown in Fig. 3. The variation of the UV-Vis spectra of the optimum concentration of SBC in paraffin oil and different concentrations of SBC in paraffin oil with time are shown in Fig. S3(a) and (b) in ESI.† The SBC additives added in paraffin oil have two main absorption peaks at 225 nm and 260 nm, and may be assigned to the n–π* transition from –OH to the aromatic ring and the π–π* transition of the aromatic ring. This confirmed the incorporation of phenolic moiety linked with the chitosan unit through the azomethine linkage.^68^ It has also been revealed from Fig. 3 and S3,† that there was no huge change in the absorbance peaks of the homogeneous mixtures of different concentrations of SBC additives in paraffin oil with time. Thus, the said homogeneous mixtures of different concentrations of SBC additives in paraffin oil were found to be stable up to 5400 s.

### Friction and anti-wear properties

**Fig. 4:**
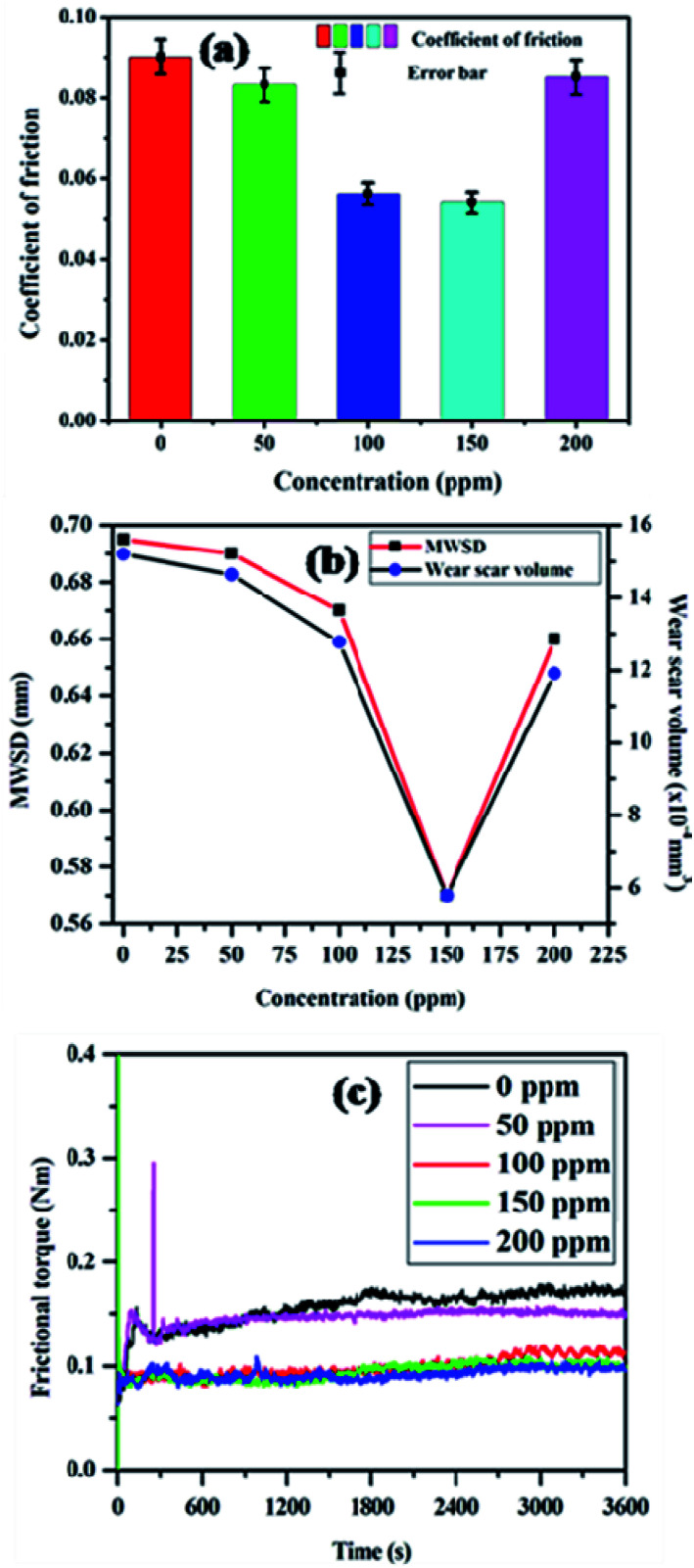
Plots of (a) concentration *vs.* coefficient of friction (b) concentration *vs.* MWSD and mean scar volume and (c) frictional torque *vs.* time obtained without and with SBC in paraffin oil at constant temperature.

**Fig. 5:**
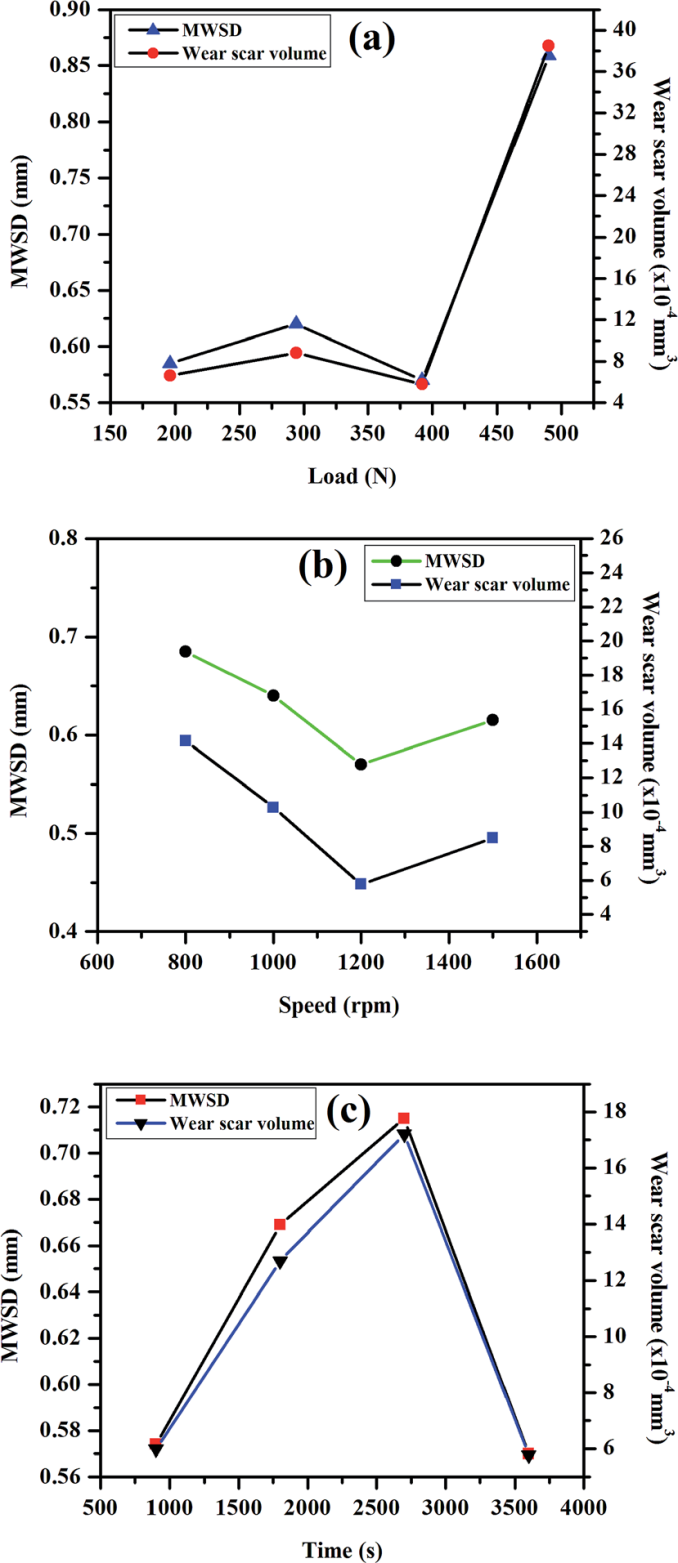
Plot of (a) load *vs.* mean wear scar diameter (MWSD) and wear scar volume, (b) speed *vs.* MWSD and wear scar volume, (c) time *vs.* MWSD and wear scar volume at a constant concentration of 150 ppm of SBC in paraffin oil and temperature.

The tribological properties of the light paraffin oil with the addition of different concentrations of the synthesized SBC additive were performed in a four-ball tester according to the ASTM D4172A (normal load of 392 N, speed of 1200 rpm, 75 °C temperature and time duration of 3600 seconds). All the plots, such as the coefficient of friction *vs.* concentration, speed, load, and time, derived from the results obtained from the four-ball tester are presented in Fig. 3, whereas the subsequent results are tabularized in Table 1. The plot of the coefficient of friction *vs.* concentration in Fig. 4(a) revealed that it decreases with the increase in the concentration of the additive from 0 ppm to 150 ppm and decreases after 200 ppm. It showed that 150 ppm of SBC additive in paraffin oil is the optimum concentration exhibiting the minimum coefficient of friction, *i.e.*, 0.0539. It was also observed that the coefficient of friction escalated when the concentration was more than the optimum concentration. The obtained outcomes can be explained based on the tribo-film formation on the steel ball. Upon the addition of 50 ppm of SBC, there was a small decrease in the coefficient of friction as compared to that of the bare paraffin oil. This may be attributed to the formation of a protective tribo-film on the exposed steel ball. Further increase in the concentration may cause an increase in the tribo-film formation with greater surface coverage. Thus, there was a simultaneous decrease in the coefficient of friction with increasing concentration. The coefficient of friction decreased up to limiting concentration in which the adsorption of the additive molecules in the frictional track was the maximum. Up until 150 ppm, the tribo-film formation on the steel surface attained saturation but at higher concentrations, there was the possibility of the agglomeration of the SBC. This was expected because chitosan and its derivatives have a tendency toward agglomeration.^66,69^ It can therefore be said that SBC at higher concentration leads to the formation of a less stable tribo-film between the two sliding surfaces during the motion, thus showing a higher coefficient of friction. The other most important tribological parameters are MWSDs and the wear scar volume of worn-out steel balls, which have also been measured and the results are tabulated in Table 1, while the obtained plot with the variation in the concentration of SBC added in lube oil *versus* MWSD and wear scar volumes have been shown in Fig. 4(b). From both Table 1 and Fig. 4(b), it was observed that at a concentration of 150 ppm of SBC a comparatively small scar was generated, which gave the lowest MWSD and wear scar volume as compared to that due to other concentrations. It was found that the MWSD and wear scar volumes at 150 ppm concentration of SBC were 0.57 mm and 5.78 × 10^−4^ mm^3^, respectively. Thus, the optimum concentration of SBC to show the minimum coefficient of friction was 150 ppm. Based on these outcomes, the lubricant formulation was prepared with the optimum concentration of SBC additive (*i.e.*, 150 ppm) in paraffin oil and it was tested in the four-ball tester by varying the normal load (196 N, 294 N, 392 N and 490 N), speed (800 rpm, 1000 rpm, 1200 rpm and 1400 rpm) and runtime (900 s, 1800 s, 2700 s and 3600 s) according to ASTM D4172A standards. All the obtained results are tabulated in Tables 2–4, while, the corresponding graphical representations have been presented in Fig. 5. From Fig. 5(a)–(c), both the MWSD and mean wear scar volume were less for the 392 N normal load, 1200 rpm speed and 3600 s run time. From Table 3, the coefficient of friction increased irregularly with the increase in load. It showed an increase up to 294 N, decreased at 392 N and again increased after the application of the 490 N normal load.

**Table 1:** Tribological parameters obtained for different concentrations of SBC additive in paraffin oil

Concentration of additive (ppm)	Coefficient of friction	Mean wear scar diameter (mm)	Wear scar volume (×10^−4^ mm^3^)
00	0.0887	0.70	15.20
50	0.0831	0.69	14.64
100	0.0561	0.67	12.77
150	0.0539	0.57	5.78
200	0.0850	0.66	11.91

**Table 2:** Calculated tribological parameters obtained for 150 ppm of SBC in paraffin oil with the variation of load

Load (N)	Co-efficient of friction	Mean wear scar diameter (mm)	Wear scar volume (×10^−4^ mm^3^)
196	0.0441	0.585	6.61
294	0.0642	0.620	8.83
392	0.0539	0.570	5.78
490	0.0879	0.859	38.48

**Table 3:** Calculated tribological parameters obtained for 150 ppm of SBC in paraffin oil with the variation of speed

Speed (rpm)	Co-efficient of friction	Mean wear scar diameter (mm)	Wear scar volume (×10^−4^ mm^3^)
800	0.0584	0.685	14.15
1000	0.0724	0.640	10.29
1200	0.0539	0.570	5.78
1400	0.0490	0.615	8.49

The reason behind this is that with the increase in the normal load the tribo-film created on the metal surface gets damaged, and this allows the steel balls to come in contact with each other, thus leading to wear. The results obtained by varying the speed on the optimum concentration of SBC additive are tabulated in Table 4 and it showed a decreasing trend in the coefficient of friction. The variations of MWSD and wear scar volumes with speed are shown in Table 4 and the corresponding plots are shown in Fig. 5(b). From Fig. 5(b), the nature of the curves for speed *vs.* MWSD and wear scar volume also decreased initially, to 1200, and increased thereafter. This may be attributed to the fact that the spreading out of the tribo-film decreases the possibility of wear on the surface of the steel balls at lower as well as higher speeds of operation. This indicates that the added SBC works well at 1200 rpm. Similarly, the experimentally obtained results and the corresponding plots for MWSD and wear scar volumes with the variation in run time are presented in Table 4 and Fig. 5(c), respectively. They show the initial increase in the MWSD and wear scar volume up to 2700 s and an abrupt decrease after exceeding 2700 s of exposure. The nature of the curves revealed that the wear always increases with runtime and this is always evident since as the time increases, the contact time also increases, which in turn increases the wear on the surface of the steel balls. The coefficient of friction was found to be 0.0539 at 3600 s run time. This revealed the achievement of the minimum value of MWSD and wear scar volume of 0.570 mm and 5.78 × 10^−4^ mm^3^ at 2700 s, respectively. This observation suggests that when sufficient time is given to the lubricant additives, it can easily form a tribo-film on the steel ball surface. The expansion of the tribo-film decreases the possibility of wear on the surface of the steel balls. The plots of frictional torque *vs.* time with the variation of (a) load and (b) speed at a constant concentration for SBC in paraffin oil and constant temperature are presented in Fig. 5. It revealed that the frictional torque is nearly constant for the optimum concentration of SBC in lube oil at 392 N load and 1200 rpm speed for the 3600 s experimental runtime.

**Table 4:** Calculated tribological parameters obtained for 150 ppm of SBC in paraffin oil for different run times

Time (s)	Co-efficient of friction	Mean wear scar diameter (mm)	Wear scar volume (×10^−4^ mm^3^)
900	0.0668	0.574	5.99
1800	0.0720	0.669	12.69
2700	0.0620	0.715	17.21
3600	0.0539	0.570	5.78

### Rheology study

The rheological parameters, such as viscosity and shear stress, were determined for the different concentrations of SBC in paraffin oil at room temperature (∼25 °C) and are tabulated in Table 5. It was observed that with the addition of low concentrations, *i.e.*, up to 150 ppm of SBC, to the paraffin oil, there was a change of ±0.004 in viscosity and ±0.4 in shear stress at room temperature. It can be said that the addition of SBC showed no effect on the viscosity up to 150 ppm, and a huge change at greater concentrations. At elevated temperatures (herein 75 °C), the viscosity as well as the shear stress of the samples were reduced. The obtained viscosity and shear stress were more or less the same for 150 ppm of SBC in paraffin oil as compared to other concentrations.

**Table 5:** Values of viscosity and shear stress for different concentrations of SBC in paraffin oil at 25 °C and 75 °C

Concentration of additive (ppm)	25 °C		75 °C	
	Viscosity (Pa s)	Shear stress (Pa)	Viscosity (Pa s)	Shear stress (Pa)
00	0.061	6.10	0.011	1.10
50	0.065	6.50	0.012	1.20
100	0.060	6.00	0.012	1.20
150	0.057	5.70	0.011	1.10
200	0.079	7.90	0.111	1.11

### Surface morphology

**Fig. 6:**
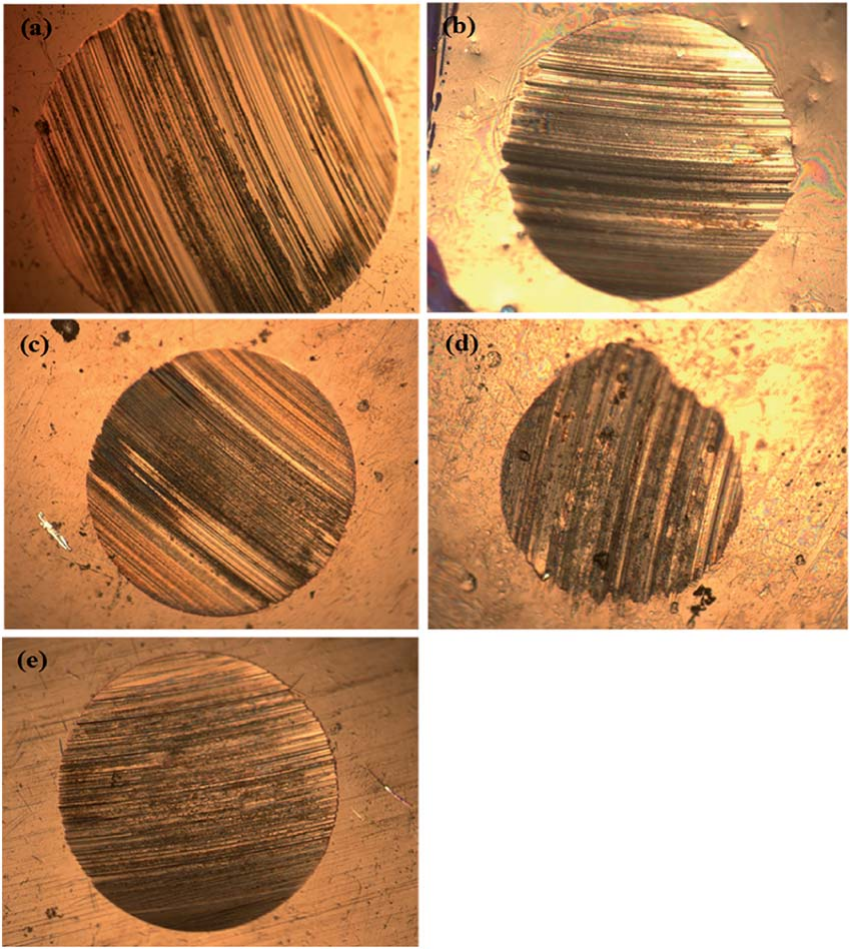
Ferrograph images of the worn out balls in (a) paraffin oil and with concentration of (b) 50 ppm, (c) 100 ppm, (d) 150 ppm and (e) 200 ppm of SBC as additive in paraffin oil.

**Fig. 7:**
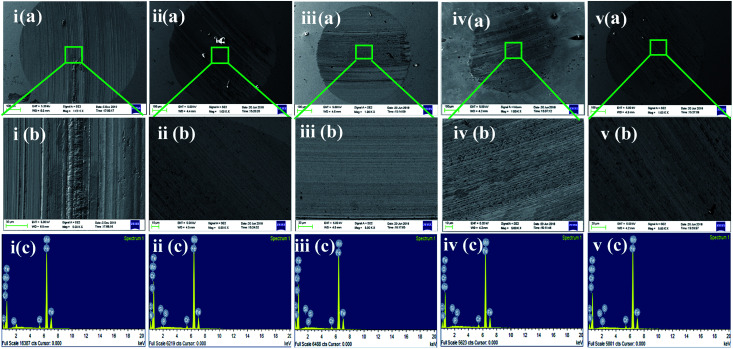
i–v (a and b) FESEM micrographs and i–v (c) elemental analysis EDX spectra of the worn out steel balls lubricated with (1) 50 ppm (2) 100 ppm (3) 150 ppm and (4) 200 ppm of SBC. [i–v (b) shows FESEM images at higher magnifications].

In the four-ball tester, the normal load was applied through the topmost ball towards the three horizontally placed balls at the bottom of the container containing the paraffin oil with and without additives. The rotating topmost ball developed scars on the surfaces of the balls placed beneath it. Ferrography images of the worn-out steel balls without and with adding different concentrations of SBC additives are presented in Fig. 6. The large wear scar made on the ball surface was subjected to only paraffin oil, and it was observed that there was a decrease in the formation of wear scar with the addition of SBC in the paraffin oil. From a close inspection of Fig. 6(d), the wear scar formed upon the addition of the optimum concentration (*i.e.*, 150 ppm) of SBC additive was the smallest as compared to those formed by the addition of other concentrations. The wear scars or the shallow portions due to the wearing of the mass from the surface of the steel balls exposed in paraffin lube oil without and with different concentrations of SBC additive were also examined using FESEM and the obtained micrographs are shown in Fig. 7.

**Fig. 8:**
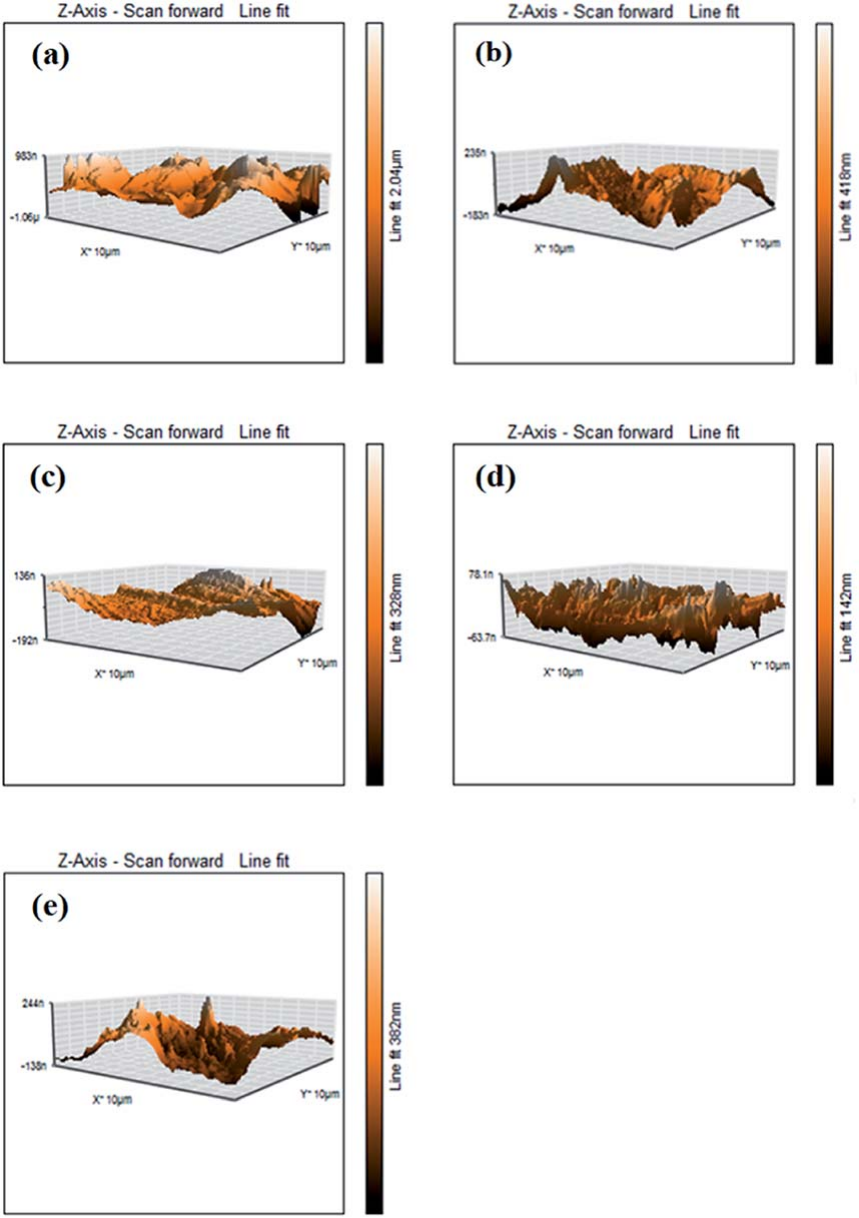
AFM micrographs of the steel balls (a) without and with the addition of (b) 50 ppm, (c) 100 ppm, (d) 150 ppm and (e) 200 ppm of SBC.

The contour fluctuations and furrows due to the wearing out of the surface are evident from the FESEM images (*vide*Fig. 7). The formed wear is adhesive wear, which is attributed to smooth wear tracks and surfaces. The furrows and the contour fluctuations varied in the images according to the measured MWSD and wear scar volumes. The low-wear scar for an optimum concentration of SBC in paraffin oil was confirmed by the FESEM micrographs and the measured MWSD and wear scar volumes. Thus, there is a crucial role played by the SBC additive in paraffin oil. In this regard, the EDX spectra of the wear scars on the balls retrieved from paraffin lube oil without and with different concentrations of SBC additive were recorded and are shown in Fig. 7. It shows the appearance of peaks for C, N, and O which reflects the presence of an organic layer formed on the surface of the chrome steel immersed in paraffin oil containing different concentrations of SBC additive. This outcome reveals the tribo-film formation on the surface of the ball, which helps to reduce the coefficient of friction and hinders the formation of wear scars by acting as a barrier. In addition to the surface morphology and elemental detection on the surface of the wear scars, the surface topography of the worn-out surfaces of the wear scars were also analyzed using atomic force microscopy. The worn surface with an area of 100.8 pm^2^ for all the samples was scanned to determine the average surface roughness and the obtained three-dimensional images are presented in Fig. 8. The obtained average surface roughness values were 50 nm, 35 nm, 15 nm and 55 nm for 50 ppm, 100 ppm, 150 ppm and 200 ppm, respectively, while chrome steel exposed to paraffin oil gave an average surface roughness of 245 nm. This suggests that the steel ball exposed to paraffin oil was degraded due to the pressure and the formed scar gave rise to high surface roughness. On the other hand, the addition of the SBC lubricant up until the optimum concentration leads to the formation of the tribo-film, thereby developing a smooth surface. The addition of the highly concentrated lubricant formulation showed a negative impact on the lubricity. The topographical results were corroborated by the outcomes obtained from the FESEM and ferrography studies.

### Density functional theory

**Fig. 9:**
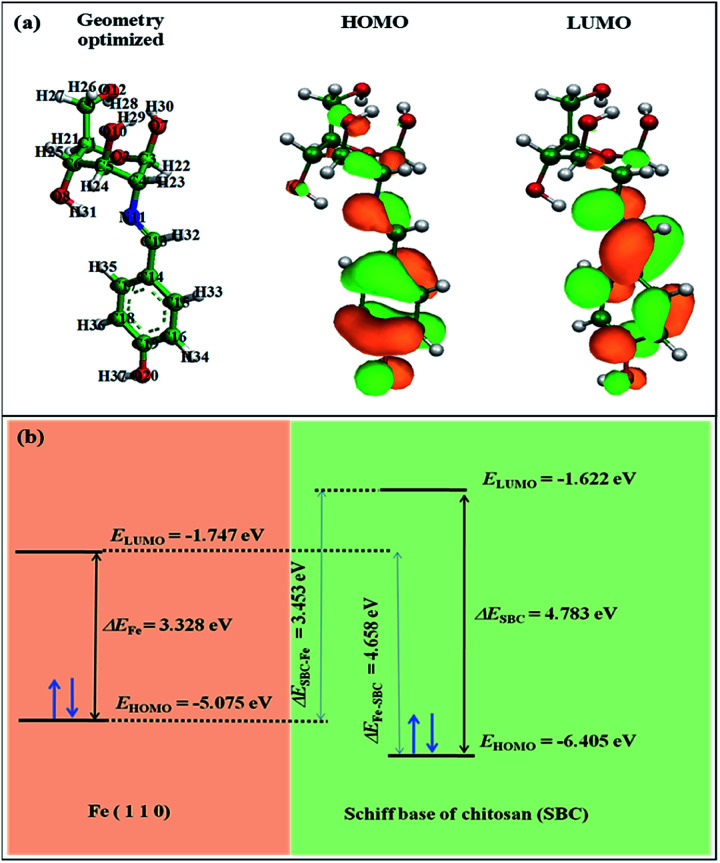
(a) The geometry optimized structure, and the HOMO and LUMO of SBC. (b) Schematic diagram showing the HOMO–LUMO energy gaps.

The obtained equilibrium geometrical structure, the electron density plot of the frontier molecular orbitals *viz.* the highest occupied molecular orbital (HOMO) and lowest unoccupied molecular orbital (LUMO) of the monomer unit of the Schiff base of chitosan (SBC) are shown in Fig. 9. The geometrical parameters of SBC are tabulated in Table S2 in the ESI.† It can be said that despite the incorporation of the 4-(iminomethyl)phenol moiety into the d-glucosamine unit of chitosan, the geometry of the structure is optimized as reflected from the obtained unaltered geometrical parameters such as bond lengths, bond angles and torsion angle of the optimized forms of SBC as shown in Table S2 in ESI† and Fig. 9(a). The HOMO region of the SBC molecule as shown in Fig. 9(a) is distributed to almost the entire molecular skeleton with the electron density mostly located in the aromatic benzene ring through the azomethine linkage to the heteroatoms of the d-glucosamine unit, while the LUMO of the molecule is mostly distributed in the aromatic benzene ring and azomethine linkage. These electron-dense sites of SBC are expected to participate in the electron sharing or interaction with the metal surface atom, thus facilitating the formation of the organic tribo-film. The energy and energy difference of HOMO (*E*_HOMO_) and LUMO (*E*_LUMO_) of SBC monomer have also been calculated and are presented in Table 6.

**Table 6:** The quantum chemical parameters obtained from DFT analysis

Molecule	*E*_HOMO_ (eV)	*E*_LUMO_ (eV)	Δ*E* (eV)	Δ*E*_SBC–Fe_ (eV)	Δ*E*_Fe–SBC_ (eV)	*I* = −*E*_HOMO_ (eV)	*A* = −*E*_LUMO_ (eV)	*χ* (eV)	*η* (eV)	*σ* (eV^−1^)	Δ*N*_110_
Fe_5_^a^	−5.075	−1.747	3.328	—	—	—	—	—	—	—	—
SBC	−6.405	−1.622	4.783	3.453	4.658	6.405	1.622	4.013	2.391	0.418	0.168

a The values for Fe_5_ are taken from Huang *et al.*^70^

Since the constituent iron atoms in the steel ball are greater than ∼97%, the study of the iron atoms was considered for simplicity, although the contributions of the other constituents have not been considered. According to Huang *et al.*,^70^ the iron has a body-centred cubic structure with the Fe (110) stable lattice plane in its metallic state. The reported values of *E*_HOMO_ and *E*_LUMO_ for the Fe (110) plane comprised of a cluster of five iron atoms are −5.075 eV and −1.747 eV, respectively.^66^ The energy gap, Δ*E*_SBC–Fe_, between *E*_LUMO_ of the SBC additive (*E*_LUMO(SBC)_) and *E*_HOMO_ of the iron surface *E*_HOMO(Fe)_, as well as the energy gap Δ*E*_Fe–SBC_ between *E*_HOMO_ of iron surface and the energy of the SBC additive *E*_LUMO(SBC)_ have been determined as eqn (12) and (13), respectively, and the corresponding outcomes have been tabulated in Table 6.

12 Δ*E*_SBC–Fe_ = *E*_LUMO(SBC)_ − *E*_HOMO(Fe)_

13 Δ*E*_Fe–SBC_ = *E*_LUMO(Fe)_ − *E*_HOMO(SBC)_

Usually, the interactions between lubricants or lubricant additives and the metal surface take place through the interaction of the HOMO of the iron atom with the LUMO of the SBC monomer and *vice versa*. Thereby, it facilitates adsorption on the metal surface.

The energy gap (Δ*E*) for SBC is the difference between *E*_LUMO(SBC)_ and *E*_HOMO(SBC)_, as shown in eqn (14).

14 Δ*E* = *E*_LUMO(SBC)_ − *E*_HOMO(SBC)_

It was expected that the transfer of electron(s) from the HOMO of SBC could take place by facilitating the chemical bonding between SBC and Fe atoms. On the other hand, the electron(s) transfer from the HOMO of the Fe atom towards the LUMO of the SBC unit showed the tendency to form back bonding synergistically. Thus, it strengthened the bonding between SBC and Fe atoms. Generally, the adsorption of the molecule on the metal surface takes place through electron transfer occurring between the metal surface atom and the adsorbate (*i.e.*, organic molecule). In this regard, the fraction of electron transfer (Δ*N*) is a very important parameter that helps in determining whether the transfer of electrons is feasible from the molecule to the metal or *vice versa*. The Δ*N* values were calculated using the pre-determined values of electronegativity (*χ*) and global hardness (*η*) for both SBC and the Fe (110) surface.15
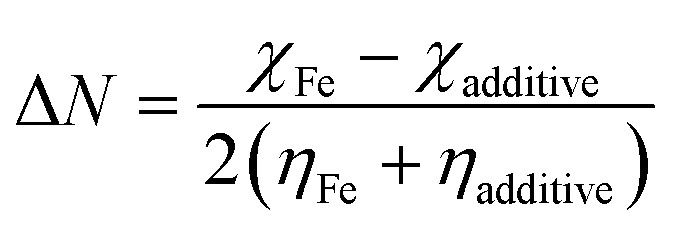
*χ*_Fe_ and *χ*_additive_ are the electronegativity of iron and the additive molecule, while, *η*_Fe_ and *η*_additive_ are the global hardness of iron and the additive molecule, respectively.

Recently, it has been preferred to use the work function (*Φ*) for calculating Δ*N* values, since it considers the electron–electron interactions in addition to the electron gas Fermi energy of iron atoms.^71–73^ Thus, the modified equation for calculating Δ*N* can be written as follows:16
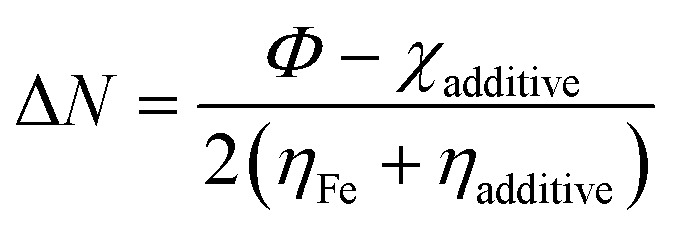
where the work function is represented as *Φ*.

The theoretical values of *χ*_Fe_ = 7 eV and *η*_Fe_ = 0 reported elsewhere in the literature^74,75^ and the *Φ* = 4.82 for Fe (110) have been used to determine the Δ*N* values.^76,77^ In this experiment, the Fe (110) surface is preferably used since it possesses a high stabilization energy as well as a highly packed structure as compared to other planes, *viz.*, Fe (111), Fe (100), *etc*. The calculated Δ*N* values have been tabulated in Table 6. Generally, it is known that Δ*N* values are positive when the transfer of electrons is taking place from the additive molecules to the metal surface atoms, and it is negative when the reverse phenomenon occurs.^78,79^ Again, as proposed by Elnga and co-workers, the electron donation capability of the molecules increases when the Δ*N* values are less than 3.6.^80^ From Table 6, the obtained Δ*N* value for the SBC additive is positive and less than 3.6. Therefore, it is expected that SBC can easily give away its electron to the vacant d-orbitals of metal surface atoms to facilitate its interactions leading to adsorption.

Furthermore, the DFT-calculated global softness (*σ*) value revealed the soft nature of the SBC additive having a *σ* value of 0.418 eV^−1^. Based on the hard and soft acid–base (HSAB) principle, the iron atoms are considered as soft centres, and it is assumed that the soft–soft interaction is mostly preferred. Hence, the interaction of SBC with iron surface atoms is feasible and it leads to the formation of a thin organic tribo-film that helps in retarding the wear of the metallic surface.

### Fukui indices analysis

The locally active nature of the atoms of a molecule acting as sites for electrophilic and nucleophilic attacks is determined using the Fukui indices (FIs) analysis.^81–83^ The maximum threshold *f*^+^_k_ and *f*^−^_k_ values are used to designate the sites responsible for the nucleophilic and electrophilic attacks on the locally active atoms, respectively.^84,85^ The locally reactive atoms of the SBC additive have been identified by FIs analysis and are presented in Table 7. It was observed that O7, O8, O10, N11, C13, C14, C15, C16, C14, C15, C16, C17, C18, C19 and C20 are the most favourable sites for both nucleophilic and electrophilic attack. On the other hand, the O2 and O12 heteroatoms act as the centres for electrophilic attack. Observation of both *f*^+^_k_ and *f*^−^_k_ values revealed that the azomethine group (–CH=N–), the aromatic π-bonds of the benzene ring and the heteroatoms (herein N and O), are the sites mostly responsible for nucleophilic attacks, as well as electrophilic attack. In the present context, it can be said that the susceptible sites for nucleophilic and electrophilic attack will share electrons with the metal surface atoms. Alternatively, the analysis of the dual descriptor (Δ*f*) where, Δ*f* = *f*^+^_k_ − *f*^−^_k_, can also be used to determine the site prone to nucleophilic and electrophilic attacks. Literature shows that if Δ*f* > 0, then the sites are considered to be nucleophilic and *vice versa*.^86,87^ In the present investigation (*vide*Table 7), it has been found that C1, O2, C5, C6, O7, O8, C9, O10, O12, C14 and O20 act as sites for electrophilic attacks, while C3, C4, N11, C13, C15, C16, C17, C18 and C19 act as sites for nucleophilic attacks. In this way, it can be said that the entire molecule can interact with the metal surface atom and is adsorbed on the metal surface.

**Table 7:** Calculated Fukui functions *f*^+^_k_, *f*^−^_k_ and Δ*f* for SBC

Atoms	SBC		
	*f* ^+^_k_	*f* ^−^_k_	Δ*f*
C1	0.002	0.012	−0.010
O2	0.004	0.018	−0.014
C3	0.007	0.007	0.000
C4	0.011	0.009	0.002
C5	0.007	0.020	−0.013
C6	0.003	0.023	−0.010
O7	0.016	0.018	−0.002
O8	0.016	0.112	−0.096
C9	0.004	0.010	−0.006
O10	0.028	0.094	−0.066
N11	0.106	0.026	0.080
O12	0.012	0.025	−0.013
C13	0.111	0.026	0.085
C14	0.037	0.040	−0.003
C15	0.049	0.030	0.019
C16	0.047	0.044	0.003
C17	0.063	0.025	0.019
C18	0.042	0.034	0.008
C19	0.073	0.045	0.028
O20	0.063	0.068	−0.005

### Molecular dynamics simulation

**Fig. 10:**
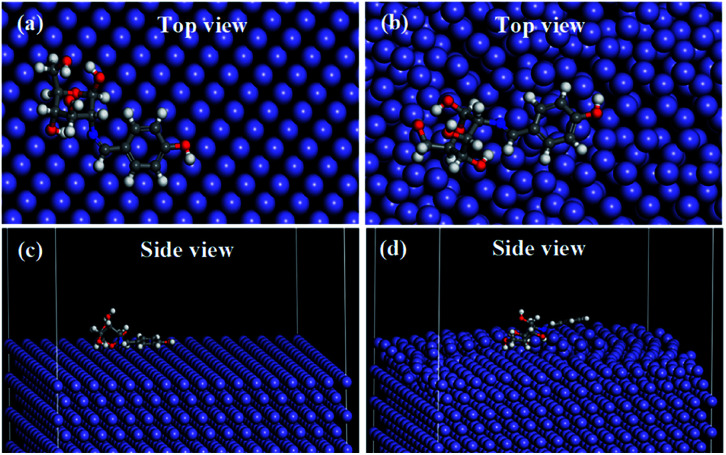
Adsorption of SBC, obtained from molecular dynamics simulation with (a and c) all the layers of Fe (110) surface fixed and (b and d) the upper two layers of the Fe (110) surface were not fixed while the other layer below it was fixed.

The molecular dynamics simulation provides significant information regarding the interactions of a molecule with metal surface atoms. The equilibrium adsorption configuration of SBC on the Fe (110) surface was obtained when the temperature and energy fluctuation curves during dynamics simulation were stabilized (*vide* Fig. S4 and S5 in ESI†). It was observed that the SBC molecule oriented itself in such a fashion that it covered a greater surface area, *i.e.* horizontally disposed on both the relaxed and fixed surface atom of Fe (110) as shown in Fig. 10.

It is evident from the side and top views of the metal surface having adsorbed SBC molecules that the aromatic benzene ring and the azomethine linkage are disposed in a parallel manner over the surface atoms. It can also be seen that most of the O heteroatoms of the D-glucosamine unit of SBC that are interacting with the iron surface atoms seem to be inclined towards the iron atoms, while some O heteroatoms are disposed away from the surface. This may be due to the non-linear configuration of the D-glucosamine unit linked with the aromatic benzene unit. Thus, it can be summarized that the major portions of SBC participate in interactions with the iron surface atoms and the simulation results gave interaction energy (*E*_interaction_) values of −574.734 kJ mol^−1^ and −489.561 kJ mol^−1^ for the fixed and relaxed surface atoms, respectively. The obtained negative *E*_interaction_ values confirmed the occurrence of interactions between SBC and iron surface atoms. The less negative *E*_interaction_ values for the surface relaxed atoms are due to the interactions among the iron atoms. Since the *E*_binding_ is the negative of *E*_interaction_, it resulted in high values for fixed surface atoms (574.734 kJ mol^−1^) as compared to that due to the unfixed surface atoms (489.561 kJ mol^−1^). Thus, the outcomes obtained from the MD simulation results suggest the highly efficient interactions of SBC with the metal surface atoms.

### Radial distribution function

According to Hansen and McDonald, the radial distribution function (RDF), *g*_AB_(*r*), is defined as the density of the particles of a system as a function of distance (*r*) as follows:^88,89^17
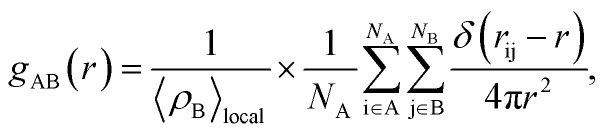
where 〈*ρ*_B_〉_local_ is the density of particle B averaged over all shells around particle A.

**Fig. 11:**
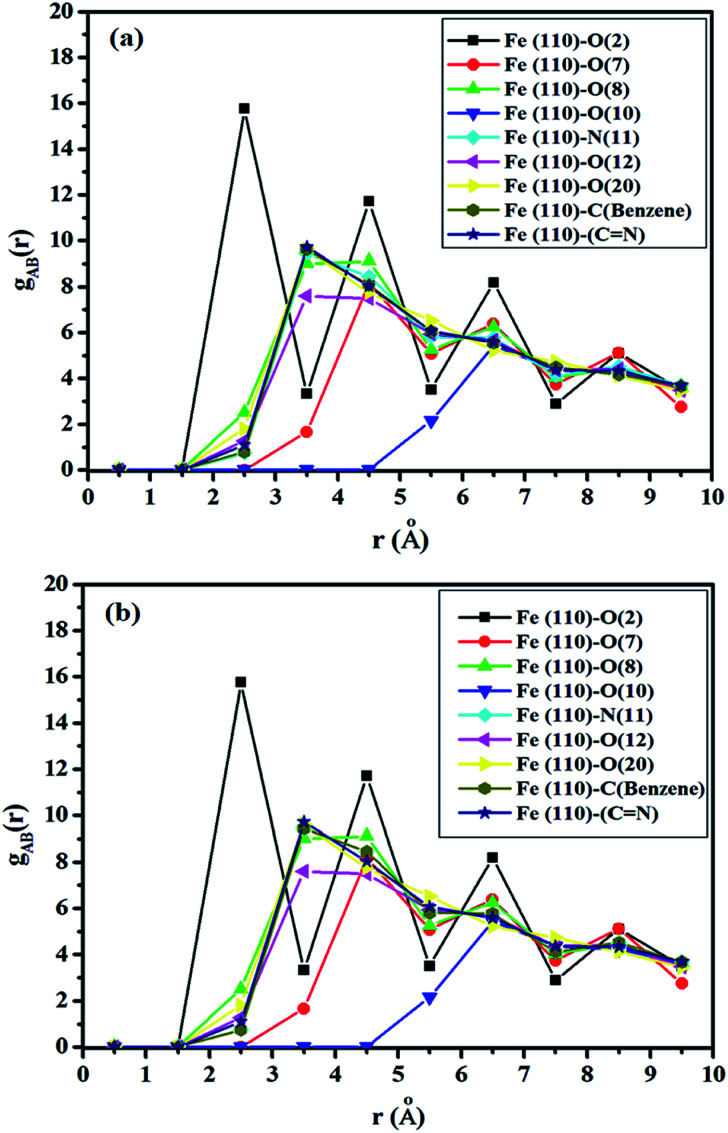
Radial distribution functions for the adsorption of SBC onto the Fe (110) surface where (a) the entire layer of the iron surface is fixed and (b) the upper two layers of the iron surface are kept relaxed and the other layers below it are fixed.

The RDF was calculated using the trajectories of MD simulation and is used to describe the density variation of atoms or groups of atoms of SBC additives as a function of the distances from the surface metal atoms. If the obtained values of the bond distance lie within 1–3.5 Å, it indicated the probable formation of chemical bonds. On the other hand, if the bond distances are longer than 3.5 Å, it suggests that the interactions are due to Coulomb and van der Waals forces, facilitating the occurrence of the physisorption of the reacting molecules with the metal surface atoms.^90,91^ The obtained RDF plot is presented in Fig. 11. The N and O heteroatoms, except for O(7) and O(10) of the SBC molecule, predominantly interact chemically with Fe (110) surface atoms as revealed from the obtained bond distance < 3.5 Å.

As shown in Fig. 11, the entire benzene moiety and azomethine linkage interact with the iron surface atoms *via* chemical bond formation. Some peaks greater than 3.5 Å were also observed. Herein, the physisorption of O(7) and O(10) atoms over the iron surface was predicted by RDF analysis. Most of the heteroatoms of SBC predominantly interact with the Fe atoms through chemical bond formation followed by physisorption.

### Adsorption mechanism of SBC on steel balls

When the synthesized SBC additive was added and finely dispersed in lube oil (herein liquid paraffin light oil) that was applied to the steel balls in the four-ball tester, SBC interacted with the metallic surface and formed a thin protective layer over it. Due to the disposition of the SBC molecules located close to the metal surface atoms, there is a great possibility for van der Waals interactions, leading to its physical adsorption on the surface of the metallic materials. This short-range distance-dependent interaction between the atoms of organic molecules and the metal surface atoms leads to the formation of a layer that is weakly adhered to the surfaces.

**Scheme 3:**
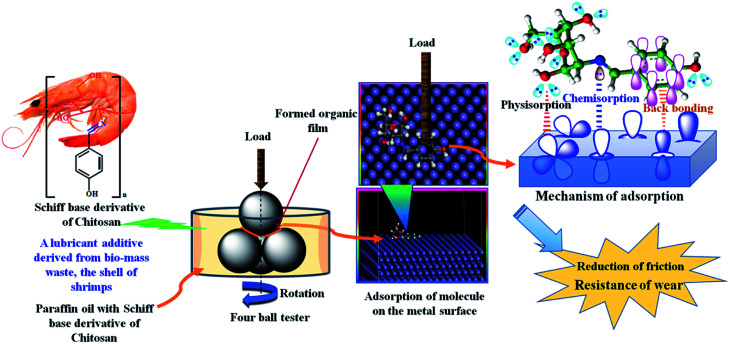
Schematic diagram showing friction reduction and wear-resistance due to the addition of SBC to paraffin lube oil.

In this way the SBC gets physisorbed on the surface of the metals. The initially physisorbed molecules further interact with the metal surface atoms and tightly adhere to the metal surface through chemical bond formation. It is evident from the molecular structure of the SBC additive that it possesses N and O heteroatoms, aromatic π-bonds and azomethine linkages as shown in Scheme 3. The main driving force behind the propensity of the adsorption of SBC on the metal surface is the lone pair(s) of electrons on N and O heteroatoms. The lone pairs of electrons from the filled p-orbitals of heteroatoms drift toward the empty d-orbitals of the metal and help in the formation of chemical bonds (*i.e.* chemisorption). Thus, there is increased electron density on metal atoms that impels the electrons from the filled d-orbitals of the metal to interact with the empty π* (antibonding) molecular orbitals of the C=C and >C=N bonds of the benzene ring and the azomethine linkage (*i.e.*, indicating the formation of back bonding). The physisorption as well as synergistic chemical bonding between SBC and metal surface atoms are the main causes for the generation of the protective tribo-film. This tribo-film minimizes the wear loss and reduces the coefficient of friction between the two interacting surfaces.

## Conclusions

A Schiff base derivative of chitosan (SBC) was synthesized and its structure was confirmed by FT-IR and CHN analysis. The SBC was found to be thermally stable up to 240 °C. Different concentrations of SBC as a biolubricant additive in paraffin oil were analyzed following the ASTM D4172A standard and it was found that the optimum concentration of the additive was ∼150 ppm. With the optimum concentration of SBC additive, the anti-wear and anti-friction properties were analyzed. The 150 ppm SBC additive in paraffin oil exhibited a load-bearing capacity of 490 N, and endured the load for up to 3600 s at 1200 revolution per minute (rpm). The coefficient of friction at the optimum concentration was reduced from 0.8 to 0.053, which may be attributed to the formed tribo-film of the SBC additive on the metal surface acting as a protective layer. The rheology study showed that the addition of SBC caused slight changes in the viscosity up to 150 ppm in paraffin oil and a huge change at greater concentrations. The FESEM and ferrography images of the wear scar revealed that 150 ppm of SBC additive is the optimum concentration for showing the desired anti-wear and anti-friction properties. The SBC additive-formed tribo-film was also confirmed from EDX analysis. Surface topography analysis of the wear scar revealed that 150 ppm of SBC additives gave rise to ∼15 nm surface roughness, while the ball exposed to paraffin oil had a surface roughness value of ∼245 nm. To validate the experimental outcomes and insightful exploration of the cause of the reduction in the wear and coefficient of friction, density functional theory and molecular dynamics simulation were employed. The optimized geometrical configuration and the electronic properties (*viz.* HOMO, LUMO, *E*_HOMO_, *E*_LUMO_, global softness, fraction of electron transfer) revealed the horizontal disposition of the active part of the molecular skeleton and the spontaneity of the adsorption of SBC additive on the metal surface, respectively. Fukui indices analysis of SBC also revealed the presence of active sites (N, O and sp^2^ C atoms from the benzene moiety) that facilitated its adsorption on the metal surfaces. The molecular dynamics simulation revealed the spontaneous interaction and the adsorption of the monomer unit of SBC on the fixed and relaxed surface of the highly packed Fe (110) plane. It showed that the monomer unit of SBC was horizontally placed on the surface of the Fe (110) plane. It can be said that the adsorption of the SBC is feasible due to the presence of the sp^2^ C atom from the benzene moiety, the azomethine linkage and the N, O-like heteroatoms. The radial distribution function analysis from the dynamics trajectories of the adsorption of SBC additive revealed that predominantly chemisorptions, followed by the physisorption of SBC on Fe (110) surface, strengthened the adherence of the organic tribo-film on the metal surface.

The results obtained from experiment and the insightful theoretical calculations and simulations, such as density functional theory, local reactivity analysis, molecular dynamics simulation and radial distribution function were in good agreement. Together, they provide a complete picture of the cause of the reduction of wear and the coefficient of friction-like tribological properties.

## Conflicts of interest

There are no conflicts to declare.

## Supplementary Material

RA-010-D0RA07011D-s001
