# Supplementary material for: Efficient tribological properties of azomethine-functionalized chitosan as a bio-lubricant additive in paraffin oil: experimental and theoretical analysis
Source: RSC Adv. 2020 Sep 10;10(55):33401–16. doi: 10.1039/d0ra07011d (PMC9056676; doi:10.1039/d0ra07011d)
Supplement: RA-010-D0RA07011D-s001 [file RA-010-D0RA07011D-s001.pdf]

Electronic Supplementary Material (ESI) for RSC Advances.
This journal is © The Royal Society of Chemistry 2020

Efficient tribological properties of azomethine functionalized chitosan as bio-lubricant additive in paraffin oil: Experimental and theoretical analysis

Manilal Murmu[a,b], Sirsendu Sengupta[a,b,‡], Ritam Pal[c,‡], Sukdeb Mandal[a,b] Naresh Chandra Murmu[a,b] and Priyabrata Banerjee*[a,b]

[a]Surface Engineering and Tribology Division, Central Mechanical Engineering Research Institute, M.G. Avenue, City Centre, Durgapur 713209, West Bengal, India.

[b]Academy of Scientific and Innovative Research, CSIR- Human Resource Development Centre, (CSIR-HRDC) Campus, Postal Staff College Area, Sector 19, Kamla Nehru Nagar, Ghaziabad 201 002, Uttar Pradesh, India.

[c]Department of Mechanical Engineering, Jadavpur University, Jadavpur, Kolkata 700032, West Bengal, India.

*Corresponding Author: P. Banerjee, e-Mail: pr_banerjee@cmeri.res.in; Website: https://www.cmeri.res.in & http://www.priyabratabanerjee.in

‡These authors contributed equally to the work.

A. According to Koopman's theorem, the ionization energy (*I*) and electron affinity (*A*) are associated with E_{HOMO} and E_{LUMO}, respectively. The magnitude of *I* and *A* is the negative of E_{HOMO} and E_{LUMO}, respectively. [S1]

$$I = -E_{HOMO} \tag{S1}$$

$$I = -E_{HOMO} \tag{S2}$$

$$\chi = \frac{I + A}{2}$$

(S3)

$$\eta = \frac{I - A}{2}$$

(S4)

B. COMPASS forcefield

The functional form of COMPASS force field is written as:[S2-S5]

$$E = E_{bond} + E_{angle} + E_{oop} + E_{torsion} + E_{cross} + E_{elec} + E_{lj} \tag{S5}$$

Where E_{bond}, E_{angle}, E_{oop}, $E_{torsion}$, E_{cross}, E_{elec} and E_{lj} are the energy contributions of bond stretching, angle bending, out of plane angle coordinates, torsion, cross coupling, electrostatic and van der Waals interactions. The terms used in equation (5) can be expanded as follows:

$$E_{bond} = \sum_{b} [k_2(b-b_0)^2 + k_3(b-b_0)^3 + k_4(b-b_0)^4] \tag{S6}$$

$$E_{angle} = \sum_{\theta} [H_2(\theta-\theta_0)^2 + H_3(\theta-\theta_0)^3 + H_4(\theta-\theta_0)^4] \tag{S7}$$

$$E_{torsion} = \sum_{\phi} [V_1[1-\cos(\phi-\phi_0)^2] + V_2[1-\cos(2\phi-\phi_0)^2] + V_3[1-\cos(3\phi-\phi_0)^2]] \tag{S8}$$

$$E_{oop} = \sum_{\chi} k_{\chi}\chi^2 \tag{S9}$$

$$E_{cross}=\sum_{b}\sum_{b'}F_{bb'}(b-b_0)(b'-b_0')+\sum_{\theta}\sum_{\theta'}F_{\theta\theta'}(\theta-\theta_0)(\theta'-\theta_0')+\sum_{b}\sum_{\theta}F_{b\theta}(b-b_0)(\theta-\theta_0)$$
$$+\sum_{b}\sum_{\phi}F_{b\phi}(b-b_0)[V_1\cos\phi+V_2\cos 2\phi+V_3\cos 3\phi]+\sum_{b'}\sum_{\phi}F_{b'\phi}(b'-b_0')[V_1\cos\phi+V_2\cos 2\phi+V_3\cos 3\phi]$$
$$+\sum_{\theta}\sum_{\phi}F_{\theta\phi}(\theta-\theta_0)[V_1\cos\phi+V_2\cos 2\phi+V_3\cos 3\phi]+\sum_{\theta}\sum_{\theta'}\sum_{\phi}k_{\phi\theta\theta'}\cos\phi(\theta-\theta_0)(\theta'-\theta_0')$$

(S10)

$$E_{elec}=\sum_{i.j}\frac{q_i q_j}{\varepsilon r_{ij}} \quad \text{(S11)}$$

$$E_{lj}=\sum_{i.j}\varepsilon_{ij}\left[2\left(\frac{r_{ij}^0}{r_{ij}}\right)^9-3\left(\frac{r_{ij}^0}{r_{ij}}\right)^6\right] \quad \text{(S12)}$$

where $b,\theta,\phi,$ and χ signifies the bond length, valence angle, torsion angle and out of plane angle, respectively.

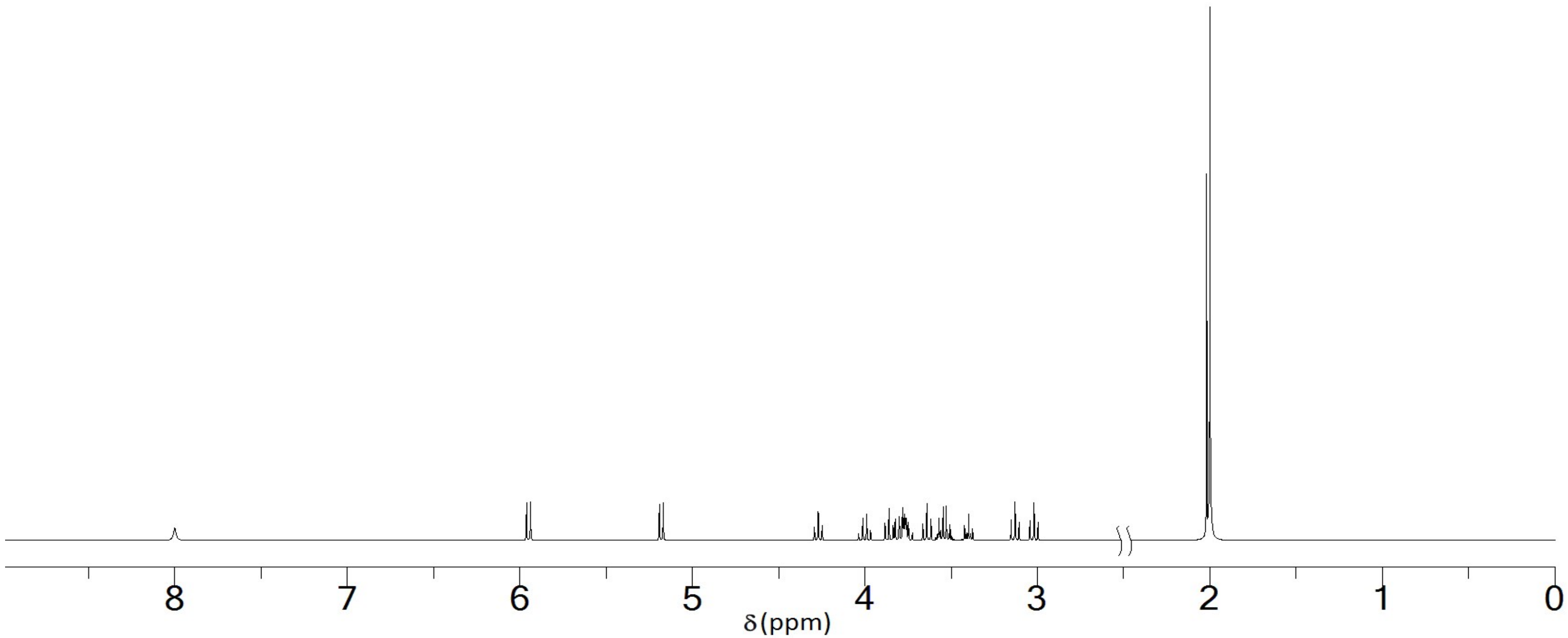

Chemical Shift (in ppm)	Proton(s)	Chemical Shift (in ppm)	Proton(s)
8.0	s, 1H, -NH –C(=O)-	3.79	d, 2H, CH (tetrahydropyran)
5.96	d, 1H, CH (tetrahydropyran)	3.76	m, 1H, CH (tetrahydropyran)
5.18	d, 1H, CH (tetrahydropyran)	3.54	d, 2H, CH (tetrahydropyran)
4.27	t, 1H, CH (tetrahydropyran)	2.02	t, 3H, $-CH_3$
4.0	dd, 1H, CH (tetrahydropyran)	2.0	brs, 4H (-OH); 2H ($-NH_2$)
3.86	m, 1H, tetrahydropyran-H		

Fig. S1(a) ^{1}H-NMR spectra of SBC in $DMSOD_6$ at 298K with chemical shifts (δ) in ppm.

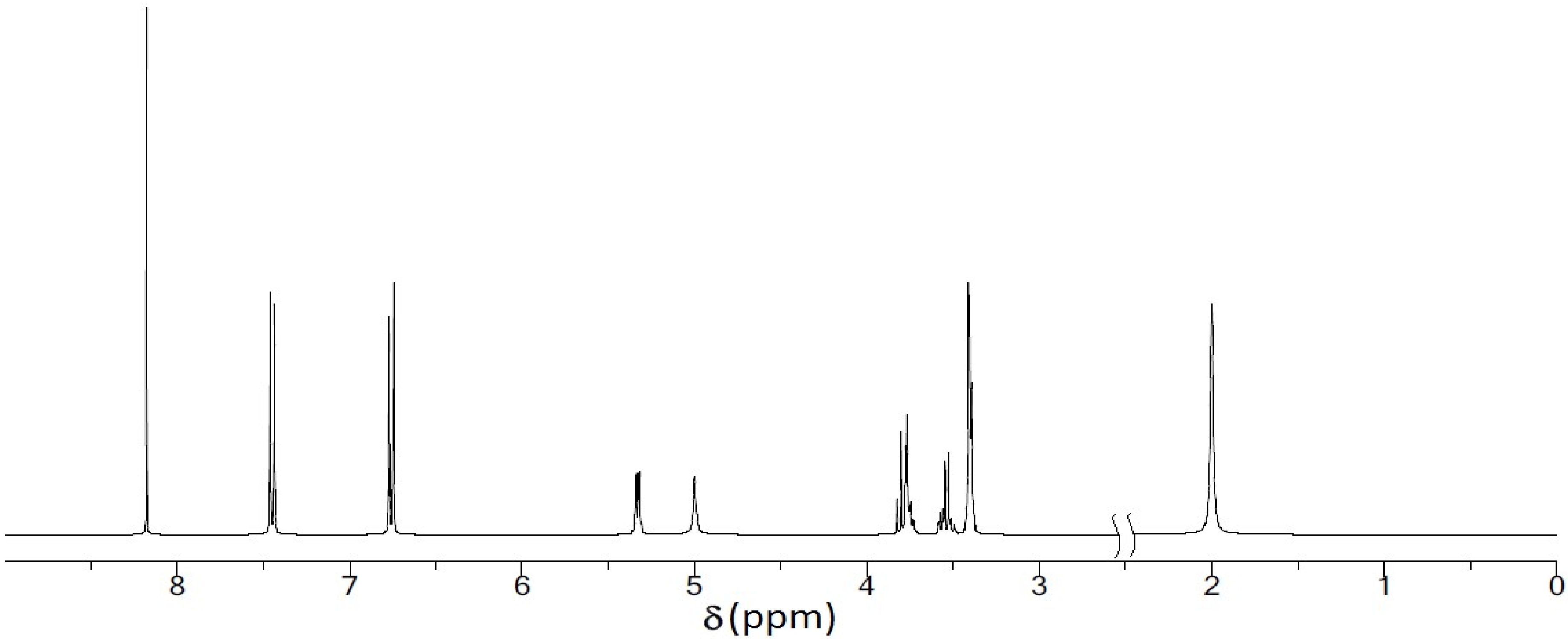

Chemical Shift (in ppm)	Proton(s)	Chemical Shift (in ppm)	Proton(s)
8.18	s, 1H, CH=N	3.4	m, 3H, CH (tetrahydropyran)
7.45	d, 2H, Ar-H	5.3	d, 1H, CH (tetrahydropyran)
6.76	d, 2H, Ar-H	3.76	m, 1H, CH (tetrahydropyran)
5.0	broad s, 1H, Ar-OH	3.54	d, 1H, CH (tetrahydropyran)
2.0	broad s, 2H, -OH	3.79	d, 1H, CH (tetrahydropyran)

Fig. S1 (b) ^{1}H-NMR spectra of SBC in $DMSOD_6$ at 298K with chemical shifts (δ) in ppm.

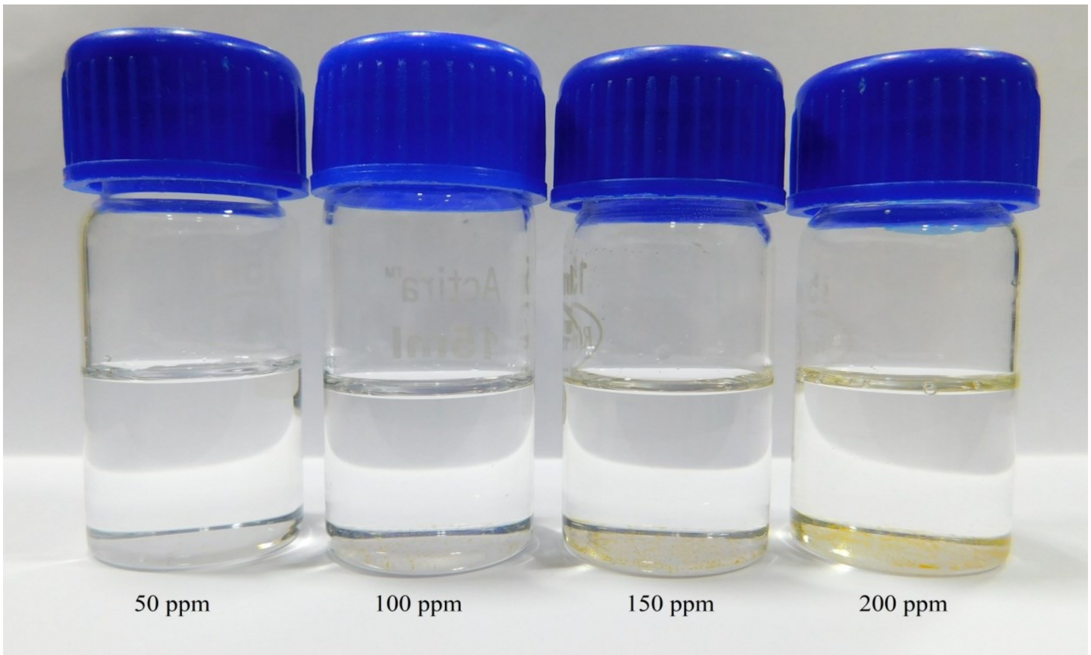

Fig. S2 Solubility of SBC indifferent concentration *viz* 50ppm, 100ppm, 150ppm and 200ppm.

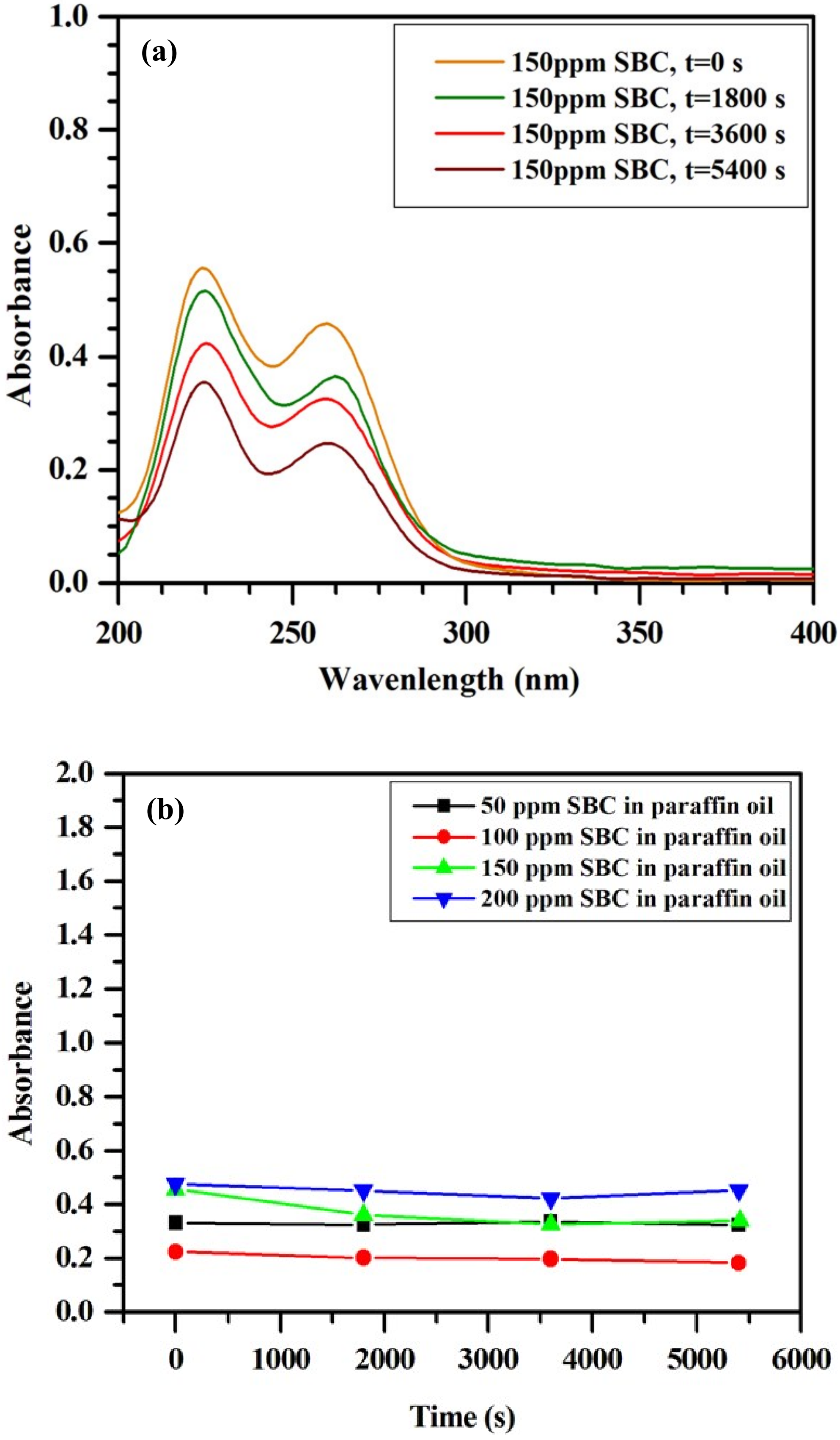

Fig. S3 Uv-Vis spectra of **(a) 150 ppm of SBC in paraffin oil and (b) different concentration of** SBC in paraffin oil with respect to time.

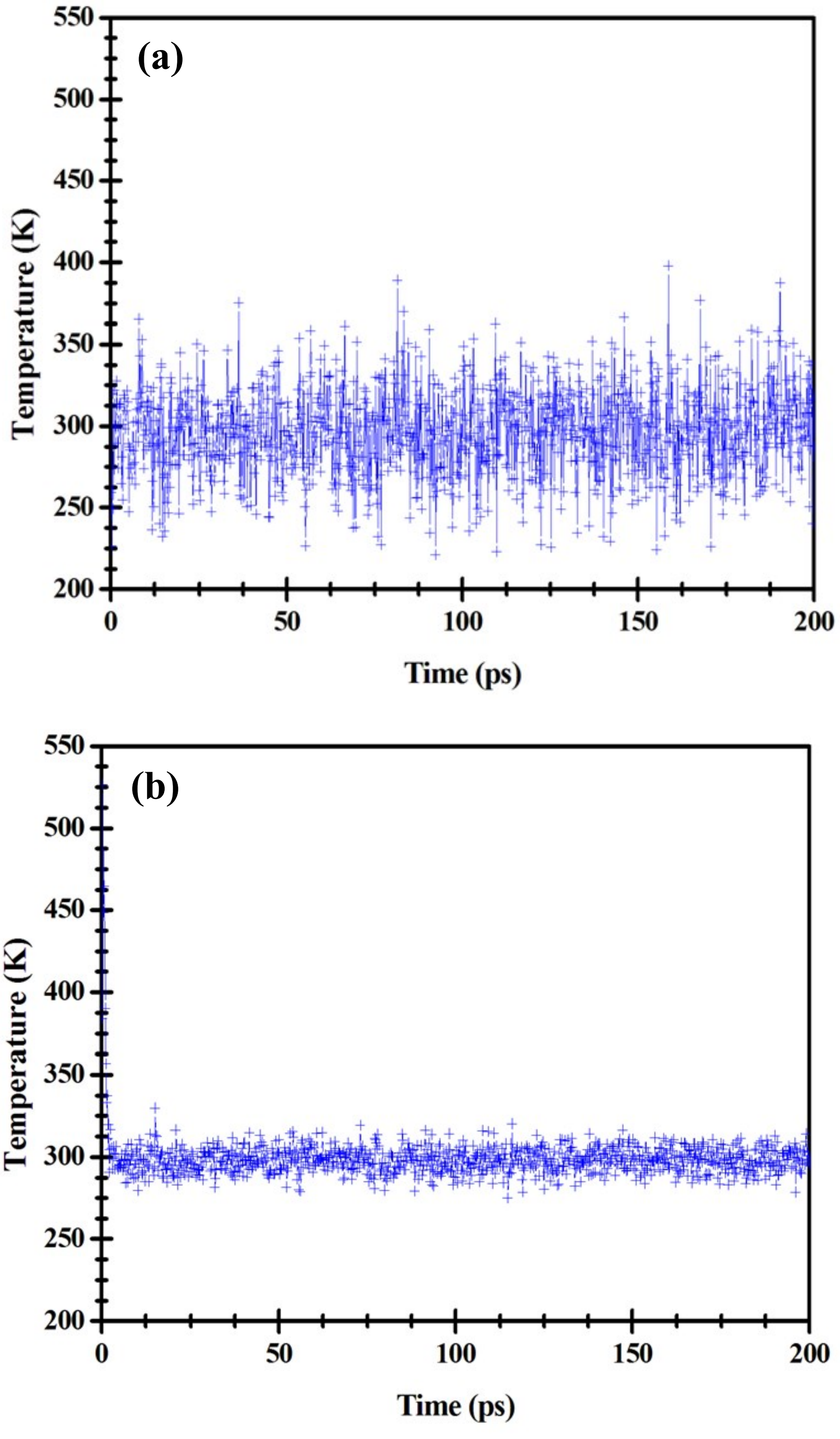

Fig. S4 Temperature equilibrium curve obtained from molecular dynamics simulation of SBC on Fe (110) surface having (a) fixed atom surface and (b) relaxed atom surface.

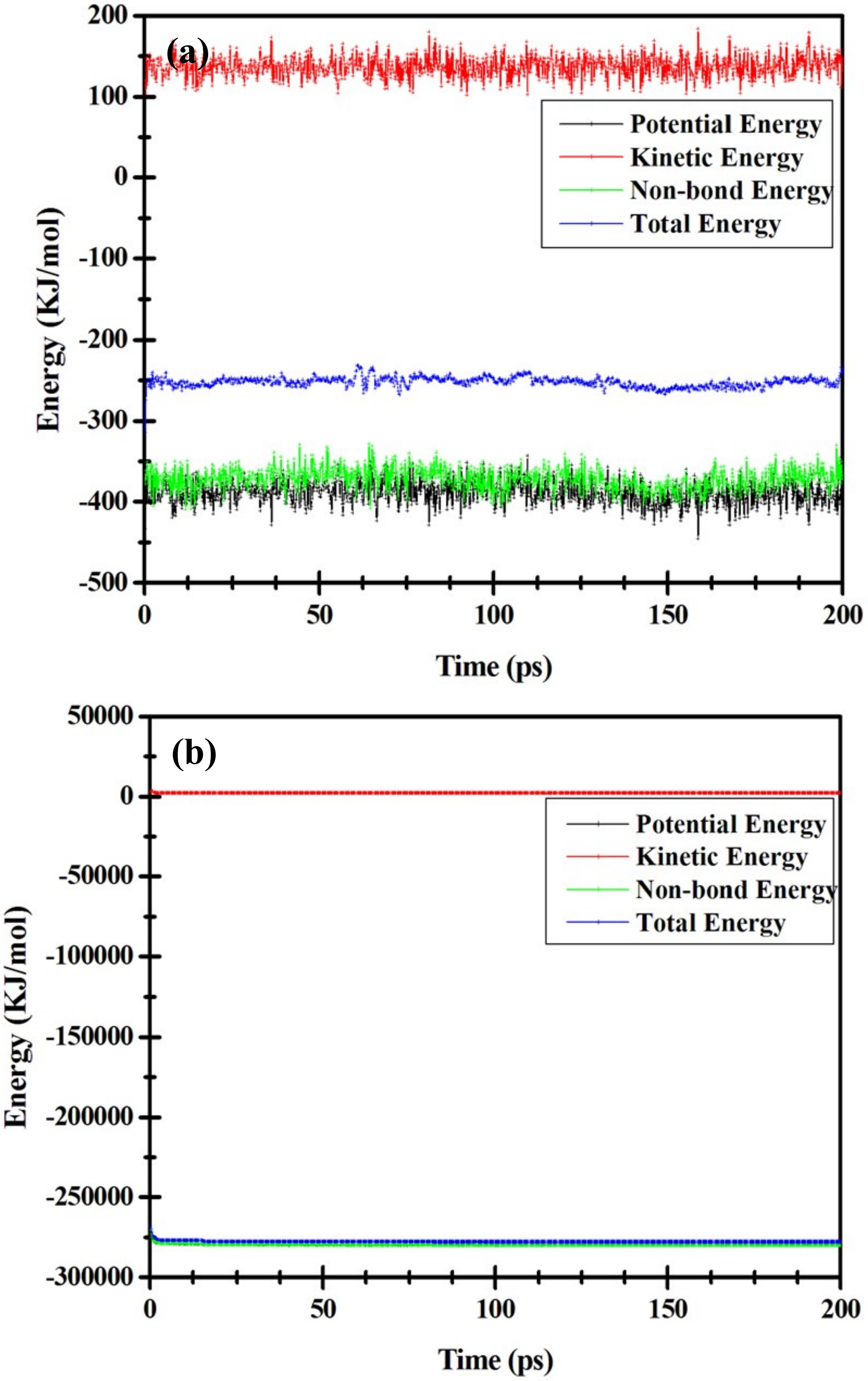

Fig. S5 Energy equilibrium curve obtained from molecular dynamics simulation of SBC on Fe (110) surface having (a) fixed atom surface and (b) relaxed atom surface.

Table S1

Geometrical parameters *viz* bond lengths (Å) , bond angles (°) and torsion angle (°) of the optimized forms of SBC.

**Geometrical parameters of SBC**					
Bond length (Å)		Bond angles (°)		Torsion angles (°)	
C1-O2	1.444	C1-O2-C3	117.27	C1-O2-C3-C4	55.76
O2-C3	1.407	O2-C3-C4	112.65	O2-C3-C4-C5	55.49
C3-C4	1.544	C3-C4-C5	109.26	C3- C4-C5-C6	52.96
C4-C5	1.550	C4-C5-C6	110.16	C4-C5-C6-C1	-50.44
C5-C6	1.553	C5-C6-C1	112.83	C5-C6-C1-C2	47.25
C6-C1	1.540	O2-C1-C9	112.46	C6-C1-C9-O12	151.23
C3-O7	1.426	C1-C9-O12	113.19	C1-O2-C3-O7	-64.98
C5-O10	1.424	O2-C3-O7	111.93	C3-C4-C5-O10	-73.68
C6-O8	1.425	C1-C6-O8	110.64	O2-C3-C4-N11	64.37
C1-C9	1.553	C3-C4-N11	109.27	C4-N11-C13-C14	179.89
C9-O12	1.431	C4-N11-C13	118.71	N11-C13-C14-C15	-177.99
C4-N11	1.455	N11- C13-C14	124.41	C13-C14-C15-C16	179.75
N11-C13	1.276	C13-C14-C15	119.17	C14-C15-C16-C19	0.06
C13-C14	1.467	C14-C15-C16	121.35	C14-C17-C18-C19	-0.06
C14-C15	1.408	C15-C16-C19	119.49	C17-C18-C19-C16	-0.09
C15-C16	1.392	C16-C19-C18	120.01	C17-C18-C19-O20	179.99
C16-C19	1.406	C19-C18-C17	119.97	–	–
C14-C17	1.408	C18-C17-C14	120.83	–	–
C17-C18	1.391	C17-C14-C15	180.36	–	–
C18-C19	1.406	–	–	–	–
C19-O20	1.364	–	–	–	–

References

S1. I. Lukovits, E. Kalman and F. Zucchi, *Corrosion*, 2001, **57,** 3-8.
S2. H. Sun, *J. Phys. Chem. B* 1998, **102**, 7338–7364.
S3. H. Sun, P. Ren and J. R. Fried*, Comput. Theor. Polym. Sci.*, 1998, **8**, 229–246.
S4. S. W. Bunte and H. Sun, *J. Phys. Chem. B* 2000, **104**, 2477–2489.
S5. A. Dutta, S. K. Saha, P. Banerjee, A. K. Patra and D. Sukul, *RSC Adv.,* 2016, **6**, 74833.
